# Depletion of the non-coding regulatory 6S RNA in *E. coli *causes a surprising reduction in the expression of the translation machinery

**DOI:** 10.1186/1471-2164-11-165

**Published:** 2010-03-11

**Authors:** Thomas Neusser, Tino Polen, René Geissen, Rolf Wagner

**Affiliations:** 1 Institut für Physikalische Biologie, Heinrich-Heine-Universität Düsseldorf, Universitätsstr 1, D-40225 Düsseldorf, Germany; 2 Institut für Biotechnologie, Forschungszentrum Jülich GmbH, D-52428 Jülich, Germany

## Abstract

**Background:**

6S RNA from *E. coli *is known to bind to RNA polymerase interfering with transcription initiation. Because 6S RNA concentrations are maximal at stationary phase and binding occurs preferentially to the holoenzyme associated with σ^70 ^(Eσ^70^) it is believed that 6S RNA supports adjustment to stationary phase transcription. Previous studies have also suggested that inhibition is specific for σ^70^-dependent promoters characterized by a weak -35 recognition motif or extended -10 promoters. There are many exceptions to this precept, showing that other types of promoters, including stationary phase-specific (σ^38^-dependent) promoters are inhibited.

**Results:**

To solve this apparent ambiguity and to better understand the role of 6S RNA in stationary phase transition we have performed a genome-wide transcriptional analysis of wild-type and 6S RNA deficient cells growing to mid-log or early stationary phase. We found 245 genes at the exponential growth phase and 273 genes at the early stationary phase to be ≥ 1.5-fold differentially expressed. Up- and down-regulated genes include many transcriptional regulators, stress-related proteins, transporters and several enzymes involved in purine metabolism. As the most striking result during stationary phase, however, we obtained in the 6S RNA deficient strain a concerted expression reduction of genes constituting the translational apparatus. In accordance, primer extension analysis showed that transcription of ribosomal RNAs, representing the key molecules for ribosome biogenesis, is also significantly reduced under the same conditions. Consistent with this finding biochemical analysis of the 6S RNA deficient strain indicates that the lack of 6S RNA is apparently compensated by an increase of the basal ppGpp concentration, known to affect growth adaptation and ribosome biogenesis.

**Conclusions:**

The analysis demonstrated that the effect of 6S RNA on transcription is not strictly confined to σ^70^-dependent promoters. Moreover, the results indicate that 6S RNA is embedded in stationary phase adaptation, which is governed by the capacity of the translational machinery.

## Background

6S RNA encoded by the gene *ssrS *is a non-coding regulatory RNA, which is wide-spread among bacteria. While most bacterial regulatory RNAs are acting at the level of translation [1,2] 6S RNA has been shown to belong to the small number of RNA molecules capable to regulate transcription [3-6]. 6S RNA from *E. coli *is transcribed together with the gene *ygfA*, whose protein product shows sequence similarity to methenyltetrahydrofolate synthetase. Despite the fact that this bi-cistronic arrangement is highly conserved among enterobacterial and g-proteobacterial ssrS transcription units its functional significance is presently not known [7,8]. The cellular concentration of 6S RNA is not constant but shows a complex regulation in response to the growth phase [9,10], reaching maximal concentrations at stationary growth. Already shortly after its discovery 6S RNA was shown to exist in the cell as a ribonucleoprotein complex [11]. Only in the year 2000 it was shown that the protein, which forms stable complexes with 6S RNA is RNA polymerase [3]. Since then, several studies have presented evidence that 6S RNA interacts specifically with RNA polymerase holoenzyme and as such inhibits transcription of a number of genes *in vitro *and *in vivo *[5,12,13]. Binding of 6S RNA occurs preferentially to the RNA polymerase holoenzyme associated with σ^70 ^(Eσ^70^) and this interaction is thought to be brought about by the particular RNA secondary structure, which is highly conserved and mimics an open promoter DNA [8,14,15]. Binding occurs to the β/β' and σ subunits of RNA polymerase and the nucleotides involved in binding have been determined by footprinting and cross-linking studies [3,5]. Moreover, in a recent investigation specific amino acids of σ^70 ^region 4.2 have been identified, which are crucial for 6S RNA binding [16].

Some uncertainty exists regarding the promoter specificity of 6S RNA-dependent transcriptional regulation. Clearly, not all promoters are sensitive to 6S RNA. During late stationary phase most of the Eσ^70 ^RNA polymerase is considered to be bound to 6S RNA and thus should generally be prevented from binding to σ^70^-dependent promoters. The mechanism of transcriptional inhibition must be more complex, however. Original observations had suggested that only a subset of σ^70 ^promoters were affected and σ^38^-dependent promoters were generally not believed to be 6S RNA sensitive. Later studies indicated that preferentially extended -10 promoters and promoters with a weak -35 consensus element were responsive to 6S RNA [3,12,15]. This observation was further substantiated by a microarray analysis performed under long-time starvation conditions [17]. Although many 6S RNA-sensitive promoters fulfil the above requirements numerous exceptions have been noted both *in vitro *and *in vivo *and in several cases results were obtained, which are inconsistent with the above supposition [5,9,17]. Obviously, the exact promoter specificity for 6S dependent genes is not all that clear and the question arises, whether the function of 6S RNA is actually confined to stationary phase-specific regulation.

To gain more insight into the complex growth phase- and promoter selectivity we have performed a global transcription analysis of wild-type and 6S RNA deficient strains using microarrays. Until now no global transcription data have been collected under exponential growth conditions. Therefore total RNA was analysed from cells grown to either mid-exponential or early stationary phase. The results reveal more than 500 differentially expressed genes, which are ≥ 1.5-fold up- or down-regulated in a 6S-dependent manner. Apparently, there is no strict correlation to a specific set of promoters, such as exponential or stationary phase-specific. The significance of the microarray data was substantiated for selected examples by qRT-PCR and quantitative primer extension analysis. The list of mRNAs with enhanced expression levels at exponential growth in the absence of 6S RNA comprise enzymes involved in guanine metabolism, a number of stress proteins and transcriptional regulators mainly involved in stress adaptation. The list of genes repressed under the same conditions in the absence of 6S RNA again includes many transcriptional regulators. During early stationary growth we find enhanced mRNA levels for several aromatic amino acid transporters, transcriptional regulators, genes involved in stress adaptation, guanine metabolism or proteins that cope with defective translation. However, the most outstanding effect during stationary growth is a concerted reduction in the expression of genes, which constitute the translational apparatus, as well as several co-transcribed genes in operons for ribosomal proteins and genes encoding translation factors. Consistent with the reduction of ribosomal protein mRNA levels in the microarray study we found a corresponding decline in the synthesis of rRNAs under stationary growth conditions using primer extension analyses. Moreover, we present evidence that the remarkable reduction in translational components is directly related to an increased basal ppGpp level in the mutant strain, probably resulting as the consequence of an imbalance in growth rate adaptation in the absence of 6S RNA. The role of 6S RNA as part of the network responsible for adjusting growth rate changes is discussed.

## Results

### Analysis of the growth phase and σ factor specificity of the 6S-dependent regulation by quantitative primer extension and qRT-PCR

**Figure 1:**
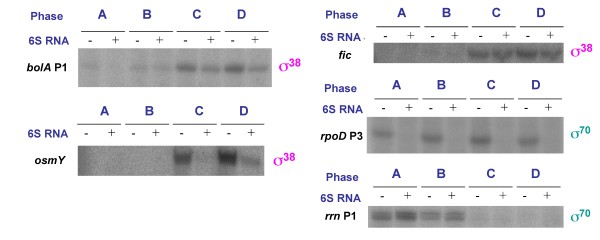
**Primer extension analysis of selected genes**. Primer extension results are exemplified for selected promoters. Total RNA was isolated from MM139 or MC4100 cells. Different growth phases for RNA isolation are indicated by A: early log (A_600 _= 0.4), B: late log (A_600 _= 1.2), C: early stationary (A_600 _= 2.7), D: stationary phase (A_600 _= 3.3). The presence of functional 6S RNA is indicated by -- or +, respectively. The predominant sigma factor specificity is indicated in brackets below the respective genes. Note that *rpoD *P3 is a minor promoter, which we have assigned as σ^70^-dependent according to its consensus sequence. Transcription of this promoter is also affected by the heat shock-specific sigma factor σ^32^[48].

Before we conducted a microarray analysis for a genome-wide transcriptional profiling we had tested a number of promoters, which differed in specificity and sigma factor dependence in order to assess the effect of 6S RNA on these selected promoters. For the analysis we used a quantitative primer extension technique [10]. The promoters were selected according to their known classification and growth phase-specificity (Additional file 1, Table S1), e.g. genes controlled by typical σ^70^-dependent promoters (*rrn *P1 or *rpoD *P3), promoters known to respond to both σ^70^- and σ^38^-RNA polymerase holoenzyme (*osmY*) and promoters preferentially transcribed by σ^38^-RNA polymerase (*bolA *P1, *fic *P). The analysis revealed that 6S RNA affects promoters independent of the characteristics of the respective promoter, indicating that there is no absolute σ factor specificity (Figure 1). We conclude that 6S-dependent regulation is apparently not strictly confined to stationary phase-specific regulation.

### Microarray analysis

In order to gain a more comprehensive view on the putative promoter specificities and genes affected by 6S RNA at the different growth phases we conducted a genome-wide microarray analysis, comparing mRNA levels in *ssrS*^+ ^and *ssrS*^- ^strains. The two strains MC4100 and MM139 used were grown at 37°C in YT media and consistent with their original characterization did not reveal measurable growth differences under the test conditions [18]. As had been observed before [10] accumulation of 6S RNA was substantial in the wild- type already in exponential phase, however, reaching about five-fold higher levels in early stationary phase. In MM139 the 6S RNA gene *ssrS *is inactivated by a *bla *insertion [18]. It has been reported previously that this *bla *insertion, disrupting the *ssrS *gene, did not give rise to the accumulation of novel RNA species containing 6S RNA sequences nor does it cause polar effects on the downstream gene *ygfA *[17,18]. In order to assess the effects of 6S accumulation on the global gene expression the RNA profiles of the 6S deficient mutant MM139 were compared with that of the isogenic wild-type MC4100 at two different growth phases: comparisons were carried out for a mid-exponential time point (OD_600 _~0.6) and the early stationary phase (OD_600 _~2.4) (Additional file 2, Figure S1).

In the DNA microarray analysis of the mid-exponential time point, 121 genes showed increased expression and 124 genes decreased expression in the *ssrS*^- ^mutant, when using 1.5-fold RNA level change as cut-off (Table 1 and 2, Additional file 3, Table S2). Notably, the *guaD-ygfQ *operon encoding guanine deaminase and a predicted transporter showed the highest significantly increased expression in the 6S deficient strain (mean values 10-fold to 18-fold, Table 1 and Additional file 3, Table S2). The guanine deaminase is involved in the salvage pathway of guanine. The *tdcABCDEFG *operon, encoding proteins involved in threonine and serine degradation, showed the second highest mRNA level changes with the leading genes *tdcABC *at the top by four-fold (Table 1, Additional file 3, Table S2). Expression of a second gene involved in purine metabolism (*add*, encoding adenosine deaminase) was also enhanced. In addition, a remarkable number of genes involved in stress adaptation also showed higher expression levels (e.g. *asr *encoding an acid shock protein and *dps *encoding a DNA protection protein showed about 2.5-fold higher expression levels in the 6S deficient strain). Likewise, genes encoding the ribosome stabilizing proteins *sra *and *yfiA *as well as the cold shock and general stress protein genes *cspA*, *uspG *and *uspF *were also up-regulated in the absence of 6S RNA. Furthermore, a number of mRNAs for transcription factors involved in environmental adaptation as well as several known and predicted/hypothetical transporters and (inner) membrane proteins showed increased expression in the *ssrS*^- ^strain (Additional file 3, Table S2).

**Table 1:** Selected genes considered as meaningful^1^, which show enhanced expression levels (≥ 1.5-fold change) during exponential phase.

Synonym	Gene^2^	Average mRNA level MM139/WT	Annotation
*b2883*	*guaD*	18.41	guanine deaminase

*b2883*	*guaD*	11.76	guanine deaminase

*b3118*	*tdcA*	4.23	transcriptional activator of *tdc *operon for biodegradation of threonine

*b1597*	*asr*	2.53	acid shock protein precursor

*b0812*	*dps*	2.34	DNA protection during starvation conditions

*b4116*	*adiY*	2.26	transcriptional activator

*b1623*	*add*	2.09	adenosine deaminase

*b1480*	*sra*	1.97	30S ribosome associated protein (S22)

*b1480*	*sra*	1.94	30S ribosome associated protein (S22)

*b4457*	*csrC*	1.93	regulatory RNA

*b1739*	*osmE*	1.90	transcriptional activator

*b2597*	*yfiA*	1.85	cold shock protein associated with 30S ribosomal subunit

*b2847*	*yqeI*	1.78	predicted transcriptional regulator

*b3361*	*fic*	1.71	stationary-phase protein, cell division

*b2707*	*srlR*	1.69	transcriptional repressor

*b3555*	*yiaG*	1.67	predicted transcriptional regulator

*b3556*	*cspA*	1.66	major cold shock protein

*b3674*	*yidF*	1.60	predicted transcriptional regulator

*b0162*	*cdaR*	1.60	transcriptional activator

*b3773*	*ilvY*	1.59	transcriptional dual regulator

*b0607*	*uspG*	1.58	universal stress protein UP12

*b1376*	*uspF*	1.57	stress-induced, ATP-binding protein

*b0460*	*hha*	1.56	modulator of gene expression, with H-NS

*b4401*	*arcA*	1.55	response regulator in two-component regulatory system with ArcB or CpxA

*b4045*	*yjbJ*	1.53	predicted stress response protein

*b2869*	*ygeV*	1.53	predicted transcriptional regulator

*b3410*	*yhgG*	1.50	transcriptional regulator

^1^Meaningful genes were selected by the following criteria: known or predicted function related to the *ssrS *transcription unit, serving common or related functions, regulatory- and stress-related functions.

^2 ^Note that some genes are represented redundantly by different oligonucleotides.

**Table 2:** Selected genes considered as meaningful^1^, which show reduced expression levels (≥ 1.5-fold change) during exponential phase.

Synonym	Gene	Average mRNA level MM139/WT	Annotation
*b0995*	*torR*	0.66	response regulator in two-component regulatory system

*b2808*	*gcvA*	0.64	transcriptional dual regulator

*b2980*	*glcC*	0.63	DNA-binding transcriptional dual regulator, glycolate-binding

*b1439*	*ydcR*	0.63	fused predicted DNA-binding transcriptional regulator

*b3319*	*rplD*	0.63	50S ribosomal protein L4

*b1526*	*yneJ*	0.62	predicted transcriptional regulator

*b3604*	*lldR*	0.62	transcriptional repressor

*b1320*	*ycjW*	0.61	predicted transcriptional regulator

*b1328*	*ycjZ*	0.60	predicted transcriptional regulator

*b3604*	*lldR*	0.57	transcriptional repressor

*b1014*	*putA*	0.54	fused DNA-binding transcriptional repressor

*b1334*	*fnr*	0.53	global transcriptional dual regulator, anaerobic growth

*b1649*	*ydhM*	0.52	predicted transcriptional regulator

*b2531*	*iscR*	0.50	transcriptional dual regulator

*b1916*	*sdiA*	0.40	transcriptional dual regulator

*b1422*	*ydcI*	0.31	predicted transcriptional regulator

*b2537*	*hcaR*	0.29	transcriptional activator of 3-phenylpropionic acid catabolism

^1^Meaningful genes were selected by the following criteria: known or predicted function related to the *ssrS *transcription unit, serving common or related functions, regulatory- and stress-related functions.

Decreased mRNA levels in the *ssrS*^- ^strain were again observed for many transcriptional regulators, including FNR, which encodes the global regulator of anaerobic growth (0.53-fold), or for the genes encoding transcriptional regulators, such as *lldR *(0.62 and 0.57-fold) or *torR*, *gcvA*, *glcC*, *putA*, *iscR*, *sdiA*, *hcaR *(Table 2). In addition, the gene *rplD *encoding r-protein L4 also shows reduced expression (Table 2, Additional file 3, Table S2).

In the DNA microarray analysis of the early stationary phase, in the absence of 6S RNA, 148 genes showed increased expression and 125 genes decreased expression, when using 1.5-fold RNA level change as cut-off (Additional file 4, Table S3). Notably, a long list of transcriptional regulators show enhanced mRNA levels in early stationary phase, including for instance Hha, YdgT and SlyA, which are all functionally related to the environmental regulator H-NS. Other regulators affected are NanR, DcuR a two-component response regulator or KdpD a two-component histidine kinase (Table 3). As already found in the exponential phase GuaD involved in purine metabolism is up-regulated. However, the strongly increased expression of the *guaD-ygfQ *operon in the *ssrS*^- ^strain observed during exponential growth was less pronounced (2.8-fold for *guaD*, Additional file 4, Table S3; 1.47-fold for *ygfQ*). Note, that the expression of this operon is known to be down-regulated during transition into stationary phase thereby weakening expression differences. Enhanced expression was also observed for genes encoding the amino acid transport systems *mtr *(tryptophane) and *sstT *(serine/threonine). In addition, the *smpB *gene shows a higher expression level (Table 3). This gene encodes the SmpB protein, which acts together with tmRNA to rescue translating ribosomes on defective mRNAs (trans-translation). One more gene of interest with enhanced expression level is *folD*, encoding a bifunctional 5,10-methylene-tetrahydrofolate dehydrogenase/5,10-methylene-tetrahydrofolate cyclohydrolase (Table 3). This enzyme is involved in one carbon metabolism and as such affects the purine synthesis [19]. Interestingly, the gene *ygfA*, co-transcribed with the 6S RNA, encodes a predicted methenyltetrahydrofolate synthetase, which would be functionally related to *folD*, indicating that the *ssrS-ygfA *operon may be linked to the purine metabolism (see Discussion).

**Table 3:** Selected genes considered as meaningful^1^, which show enhanced expression levels (≥ 1.5-fold change) during stationary phase.

Synonym	Gene	Average mRNA level MM139/WT	Annotation
*b2620*	*smpB*	3.13	SsrA-binding protein

*b2883*	*guaD*	2.78	guanine deaminase

*b3755*	*yieP*	2.94	predicted transcriptional regulator

*b3226*	*nanR*	2.41	transcriptional regulator NanR

*b0460*	*hha*	2.34	modulator of gene expression, with H-NS

*b1625*	*ydgT*	1.82	H-NS and StpA-binding protein

*b0001*	*thrL*	1.73	thr operon leader peptide

*b2865*	*ygeR*	1.72	transcriptional regulator

*b2023*	*hisH*	1.68	imidazole glycerol phosphate synthase subunit HisH

*b0529*	*fold*	1.67	bifunctional 5,10-methylene-tetrahydrofolate dehydrogenase/5,10-methylene-tetrahydrofolate cyclohydrolase

*b0080*	*fruR*	1.67	transcriptional dual regulator

*b2684*	*mprA*	1.67	transcriptional repressor of microcin B17 synthesis and multidrug efflux

*b3161*	*mtr*	1.66	tryptophan transporter of high affinity

*b1477*	*yddM*	1.66	predicted transcriptional regulator

*b4124*	*dcuR*	1.65	response regulator in two-component regulatory system

*b4212*	*ytfH*	1.64	predicted transcriptional regulator

*b3863*	*polA*	1.63	DNA polymerase I

*b4393*	*trpR*	1.63	Trp operon repressor

*b3089*	*sstT*	1.61	sodium:serine/threonine symporter

*b1214*	*ychA*	1.61	predicted transcriptional regulator

*b3438*	*gntR*	1.55	transcriptional repressor

*b0506*	*allR*	1.54	transcriptional repressor

*b1642*	*slyA*	1.52	transcriptional regulator SlyA

*b0695*	*kdpD*	1.51	fused sensory histidine kinase in two-component regulatory system with KdpE

*b3507*	*yhiF*	1.51	predicted transcriptional regulator

^1^Meaningful genes were selected by the following criteria: known or predicted function related to the *ssrS *transcription unit, serving common or related functions, regulatory- and stress-related functions.

**Figure 2:**
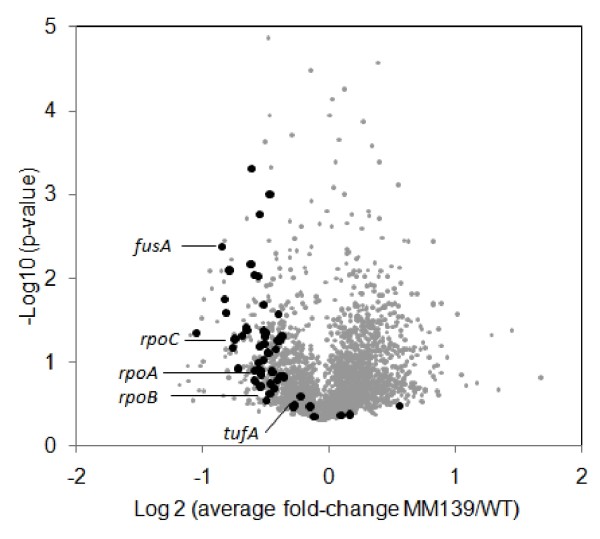
**Volcano plot of global gene expression differences**. The plot indicates global gene expression data (●) of the 6S deficient strain MM139 and WT in the early stationary phase. Expression of ~50 genes encoding ribosomal proteins and proteins involved in transcription (*rpoA*, *rpoB*, *rpoC*) and translation elongation (*tufA*, *fusA*) showed almost uniformly slightly decreased and up to 2-fold lower expression levels (●).

The most striking result of the microarray analysis, however, is the expression of a large number of genes encoding ribosomal proteins and proteins involved in transcription and translation elongation, which show uniformly the tendency of slightly decreased and up to 2-fold lower expression levels in the 6S deficient RNA strain (Figure 2, Table 4 and Additional file 4, Table S3). When a 1.5-fold RNA level change as cut-off is applied transcription of 17 individual ribosomal protein mRNAs from almost all r-protein operons are found to be reduced, comprising protein genes from both the small and the large ribosomal subunits. In addition, many genes co-transcribed within r-protein transcription units were equally reduced (Table 4, Additional file 4, Table S3). Notable examples comprise genes encoding RNA polymerase subunits *rpoB *and *rpoC*, the translation factor *fusA *(EF-G), *yfjA *(*rimM*), important for 16S rRNA processing, as well as the genes encoding primase (*priB*) or a tRNA methyltransferase (*trmD*). It should be noted that in addition to genes co-transcribed with ribosomal constituents several transcriptionally independent genes, yet functionally related to the translation process, were also found to be reduced in the *ssrS *deficient strain during stationary phase. Those genes comprise *yibK*, a predicted rRNA methylase and *lepA*, a GTP-binding protein known as a ribosomal elongation factor EF-4 (LepA), counteracting mistranslocated ribosomes [20]. Moreover, several genes encoding tRNA synthetases or tRNA modifying enzymes (*metG*, *lysS *or *tgt*) showed reduced mRNA levels.

**Table 4:** Selected genes considered as meaningful^1^, which show reduced expression levels (≥ 1.5-fold change) during stationary phase.

Synonym	Gene	Average mRNA level MM139/WT	Annotation
*b3309*	*rplX*	0.66	50S ribosomal protein L24

*b3341*	*rpsG*	0.66	30S ribosomal protein S7

*b3936*	*rpmE*	0.66	50S ribosomal subunit protein L31

*b2609*	*rpsP*	0.66	30S ribosomal protein S16

*b1892*	*flhD*	0.66	transcriptional activator FlhD

*b4202*	*rpsR*	0.65	30S ribosomal protein S18

*b3310*	*rplN*	0.65	50S ribosomal protein L14

*b3606*	*yibK*	0.65	predicted rRNA methylase

*b3296*	*rpsD*	0.65	30S ribosomal protein S4

*b2185*	*rplY*	0.65	50S ribosomal protein L25

*b4200*	*rpsF*	0.64	30S ribosomal protein S6

*b0623*	*cspE*	0.64	cold shock protein E

*b3342*	*rpsL*	0.64	30S ribosomal protein S12

*b1334*	*fnr*	0.63	DNA-binding transcriptional dual regulator of anaerobic growth

*b2569*	*lepA*	0.62	GTP-binding protein LepA

*b3231*	*rplM*	0.62	50S ribosomal protein L13

*b3340*	*fusA*	0.62	elongation factor EF-2 (S7/S12 operon)

*b1333*	*uspE*	0.59	stress-induced protein

*b3983*	*rplK*	0.58	50S ribosomal protein L11

*b3984*	*rplA*	0.57	50S ribosomal protein L1

*b3311*	*rpsQ*	0.57	30S ribosomal protein S17

*b2606*	*rplS*	0.57	50S ribosomal protein L19

*b3934*	*cytR*	0.56	DNA-binding transcriptional dual regulator

*b2608*	*rimM*	0.56	16S rRNA-processing protein

*b3636*	*rpmG*	0.55	50S ribosomal protein L33

*b0162*	*cdaR*	0.54	DNA-binding transcriptional activator

*b4201*	*priB*	0.54	primosomal replication protein N (S6 operon)

*b3988*	*rpoC*	0.54	DNA-directed RNA polymerase subunit beta' (L10 operon)

*b3987*	*rpoB*	0.53	DNA-directed RNA polymerase subunit beta (L10 operon)

*b3555*	*yiaG*	0.52	predicted transcriptional regulator

*b3703*	*rpmH*	0.45	50S ribosomal protein L34

*b1235*	*rssB*	0.11	response regulator of RpoS

^1^Meaningful genes were selected by the following criteria: known or predicted function related to the *ssrS *transcription unit, serving common or related functions, regulatory- and stress-related functions.

**Figure 3:**
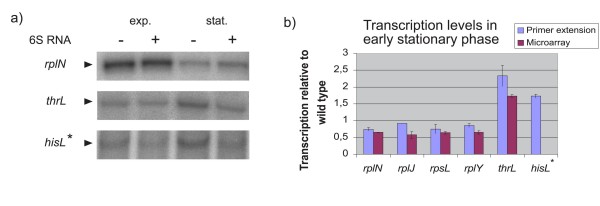
**Comparison between microarray and primer extension results**. a) Results from primer extension analyses of selected genes are presented. RNA samples were isolated at exponential or early stationary phase (exp. or stat., respectively). In b) quantitative evaluation (ratio of relative transcripts from *ssrS*^-^/*ssrS*^+ ^from two to four independent experiments) of the primer extension results for selected genes and the corresponding results from the microarray analysis for the early stationary phase are shown. * *hisL*, the leader region of the *his *operon, has not been found differentially expressed by the microarrays but is 6S RNA sensitive according to primer extension.

The expression differences observed in the microarray analysis were corroborated by quantitative primer extension analysis of the promoters of the threonine operon (*thrL*, 1.73-fold up) as well as for several ribosomal genes/operons, such as *rpsL *(*rpsG*, *fusA*), *rplJ *(*rpoB*, *rpoC*), *rplN*, *rplY*, (Figure 3). Furthermore, the *osmY*, *rpoD *and *bolA *genes have been verified by qRT-PCR analysis in two independent biological replicates, which also corroborate DNA-microarray data (no mRNA level differences, Additional file 5, Table S4).

### Transcription of ribosomal RNA is reduced in ssrS-deficient strains during stationary growth

**Figure 4:**
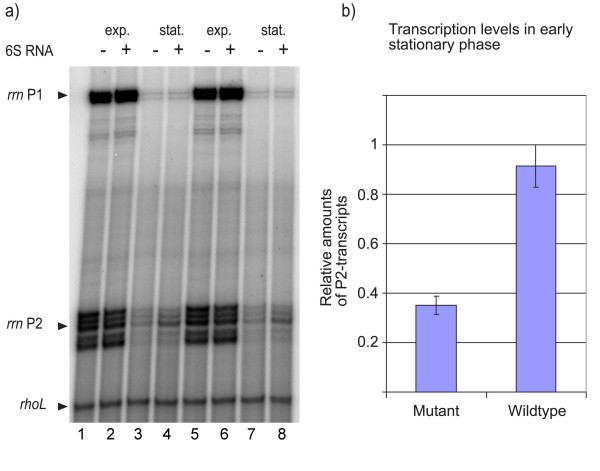
**6S RNA affects ribosomal RNA transcription at stationary growth**. a) Primer extension analysis of bulk rRNA transcription from P1 and P2 promoters. Two independent RNA samples were analyzed from *ssrS*^- ^(-) and *ssrS*^+ ^(+) cells grown exponentially (exp.) or stationary (stat.). cDNA products originating from P1 and P2 promoters are indicated. Multiple bands are resolved due to sequence heterogeneities of the rRNA leader regions from the seven different rRNA operons. The constitutively expressed *rhoL *transcript served as an internal standard for quantification. Lanes 1 and 5: RNA from *ssrS*^- ^cells at exponential growth, lanes 2 and 6: RNA from *ssrS*^+ ^cells at exponential growth, lanes 3 and 7: RNA from *ssrS*^- ^cells at stationary growth, lanes 4 and 8: RNA from *ssrS*^+ ^cells at stationary growth. b) Quantitative evaluation of P2 transcription products at early stationary phase from RNA samples of *ssrS*^- ^(Mutant) and *ssrS*^+ ^(wild-type) cells is shown. Error bars give the standard deviation of 4 independent experiments.

The down-regulation of ribosomes and translational components as the most pronounced result of the microarray analysis suggested that the presence of 6S RNA and the balance of ribosome formation during stationary phase must be linked. It is a known fact that the regulation of ribosomal biogenesis is a complex process, where the synthesis of about 60 different components has to be controlled in response to the translational demands in a timely coordinated fashion [21-23]. Ribosomal RNAs and r-proteins are regulated in a concerted way and control takes place at the transcriptional, post-transcriptional, translational and post-translational levels. Moreover, it is known that regulation of r-protein genes is dependent on the availability of free ribosomal RNA by a translational feedback mechanism [24,25]. Hence, the rate-limiting process in ribosome formation is rRNA transcription. Therefore we asked if the observed concerted reduction in r-protein mRNA levels was accompanied by a corresponding change in rRNA transcription (note that the microarrays did not include genes for stable RNA analysis). To this aim the same RNA preparation that had been employed for microarray analysis was used for determination of rRNA synthesis rates by a quantitative primer extension assay [10]. For the analysis we used a primer complementary to the leader region of all seven rRNA transcripts able to direct cDNA synthesis from P1- and P2-directed rRNA products. Due to the short half-life of the rRNA leader (~40 seconds, [26]) the measured amount of cDNA transcripts represents synthesis rates rather than accumulation of rRNAs. Results of two such representative experiments are shown in Figure 4. At exponential growth, when rRNA P1 promoters take the major load of rRNA transcription, firing almost at their maximal initiation frequency, only weak differences in the synthesis of rRNAs between wild-type and *ssrS*^- ^mutant is apparent (see also Figure 1, *rrn *P1). In contrast, a more than twofold reduction in P2-directed transcripts, representing the predominant RNA species during stationary phase, can be observed at the early stationary phase (see Figure 4b). These findings are fully consistent with the reduced r-protein levels and support the conclusion that a lack in 6S RNA indeed affects the synthesis of ribosomes under stationary growth conditions.

### The basal ppGpp level is enhanced in 6S RNA deficient strains

**Figure 5:**
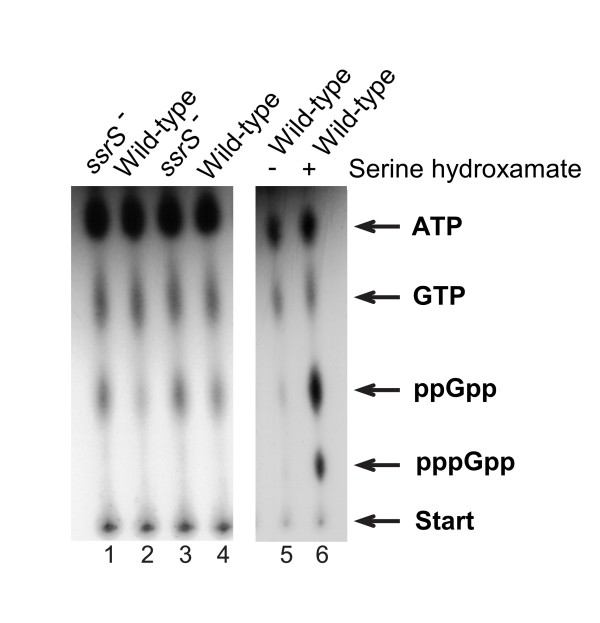
**During stationary growth the basal ppGpp level is increased in 6S RNA deficient strains**. NTPs extracted after *in vivo *labelling from two independent experiments were separated by thin layer chromatography. Lanes 1 and 3: extracts from *ssrS*^- ^strain, lanes 2 and 4: extracts from the wild-type MC4100, lanes 5 and 6: extract from the *relA*^+ ^strain MG1655. The sample in lane 6 had been treated with serine hydroxamate to induce the stringent control in order to produce high levels of ppGpp and pppGpp, which served as mobility markers.

Regulation of ribosome synthesis and most of the translational components critically depends on the concentration of the regulatory effector molecule ppGpp [22], whose concentration shows a linear inverse correlation to the rRNA synthesis rates over a wide range of growth conditions [27]. Furthermore, ppGpp plays a major role during the rapid down-regulation of rRNA synthesis at the end of the exponential phase, when cells enter stationary growth conditions [28,29]. During this transition the major load of rRNA transcription is changed from P1 to P2 promoters. Generally, rRNA P1 promoters are considered to represent the main regulatory target for ppGpp, yet the P2 promoters have also been shown to respond to changes of the effector molecule, although less dramatically [30-32]. We wished to know, therefore, whether the observed decline in rRNA synthesis is also reflected in a concomitant change of the basal ppGpp level in the *ssrS*^- ^strain. For this purpose we determined the concentration of the effector nucleotide ppGpp in wild-type and *ssrS*^- ^strains. Cells were grown under the same conditions and to the same optical density as for the microarray analysis. ^32^P labelling, isolation of nucleotides, separation by thin layer chromatography and visualization of ppGpp was performed as described in the Experimental section. Figure 5 shows the results from two independent experiments, where the ppGpp concentrations had been determined during early stationary growth. Clearly, the spots representing ppGpp are significantly enhanced in the samples extracted from the *ssrS*^- ^strain. Quantitative evaluation of the spot intensities (intensity ratios ppGpp/ppGpp+GTP) revealed 38% higher basal ppGpp concentration in the absence of 6S RNA. This increase in the effector nucleotide is fully consistent with the reduced rRNA synthesis in the *ssrS*^- ^strain and also explains the reduction in the translational components detected in the microarray analyses. We conclude therefore that the observed down-regulation of translational components during stationary phase is likely the result of an increased basal level of the regulatory compound ppGpp in response to the lack of 6S RNA. Together the results support the view that ppGpp compensates the defect in growth adaptation normally mediated by 6S RNA (see Discussion).

It should be noted that the increase in the basal ppGpp concentration is mediated by an altered activity of SpoT and does not depend on the presence or absence of RelA because the same result was obtained no matter if *relA*^+ ^or *relA*^- ^strains deficient in 6S RNA were compared (data not shown).

## Discussion and Conclusions

### 6S RNA-mediated differential expression is not restricted to special promoter classes

Given the respectable number of differentially expressed genes during exponential growth one must put into question the view that 6S RNA is a strictly stationary phase-specific regulator. Our analysis clearly shows, that not only σ^70^-dependent genes are inhibited by 6S RNA, when cells enter stationary phase, but also other types of promoters are under 6S control.

It is generally difficult to assess promoter specificity directly from microarray data since this technique measures the steady state concentration of mRNAs, which is not necessarily identical with the transcriptional activity of the respective promoters. Moreover, many genes are directed by more than one promoter, often with divergent specificities and many are additionally modulated by different transcription factors. A large number of genes are also subject to attenuation or antitermination, making an assignment of the transcript level with a distinct promoter very difficult. An unambiguous correlation between the genes identified as up- or down-regulated, based on microarray data, with the activity of the responsible promoter is therefore only possible for a limited number of cases. Nevertheless, when we analyzed the published promoter sequences of the differentially expressed genes presented in Tables 1, 2, 3 and 4 we found no clear correlation with specific promoter classes, such as σ^70^-dependent with weak -35 element or σ^70 ^extended -10 promoters [17]. In fact, quite a variety of promoters were represented according to [33], including σ^32^, σ^38^, σ^54 ^and σ^70 ^promoters of different specificity (Additional files 3 and 4, Table S2 and S3). This observation is true for both up- and down-regulated genes. In summary, our data does not exclude a preference in the regulation of σ^70^-dependent promoters with weak -35 element or σ^70 ^extended -10 promoters but it clearly shows, that many other promoters, including σ^38^-dependent stationary phase-specific promoters, are also under 6S RNA-dependent control. Our data do not disagree with the view that 6S RNA regulates transcription by binding to RNA polymerase and competing with DNA promoters. Although we cannot define absolute specificity rules our results demonstrate that promoters with variable σ factor dependence are also regulated. This fact has to be considered, when the molecular mechanism of transcriptional inhibition should be explained.

Compared to the microarray analysis the primer extension technique provides a much better correlation of transcript levels and promoter classes because transcription start sites are directly mapped. Therefore, the effects of 6S RNA on variable promoter classes presented in Figure 1 represent more reliable information. Together, the primer extension results basically support the conclusions from the much broader microarray data. A notable difference is apparent for the *osmY *gene (Figure 1 and Additional file 5, Table S4), indicating that promoter activity and mRNA accumulation are not identical in this case.

### Comparison of the results obtained in this study with a previously performed transcriptome analysis

The fact that there is only very limited coincidence between the genes showing altered expression ratios in this study and a similar investigation published recently [17] is not too surprising, considering the fact that in both studies very different growth situations were compared. While in the latter study wild-type and 6S deficient strains were compared after long-term stationary growth (16 h), which are almost metabolically silent, already adapted to zero growth and in a state of maintenance the results in this investigation were obtained by comparison of cells, which had just entered the stationary phase and were just about to adapt to the new growth conditions. We like to emphasize that no global analysis was reported for exponentially growing cells before.

### Potential function of 6S RNA during exponential growth

According to the microarray analysis a respectable number of genes is up-regulated during exponential growth, when the 6S RNA concentration in the wild-type is not maximal. Among those genes we found 14 coding for transport proteins, about the same number of transcriptional regulators, 6 stress-related proteins and 2 important enzymes involved in the metabolism of purines (Table 1 and Additional file 3, Table S2). As it is generally true for microarray data this notable increase in the mRNA levels of functional important genes in the absence of 6S RNA during exponential growth may either indicate a direct repression of these genes by 6S RNA or an indirect functional compensation due to the lack of 6S RNA. In any case, this finding suggests that there are very likely additional functions of 6S RNA different from down-regulation of certain Eσ^70^-dependent genes during stationary growth as has been proposed previously [34]. Moreover, many transporters (~20), an equal number of regulators, including two component systems, also showed reduced expression levels during exponential growth, underlining the assumption of a functional relevance of 6S RNA during exponential growth.

Interestingly, the mRNA level for *rplD *(ribosomal protein L4) is also reduced during exponential growth, suggesting a gradual regulation of the r-protein transcription units, which already initiates before cells enter stationary phase (see below).

### Functional importance of differentially expressed genes during stationary phase

The most interesting result of this study is the striking coincidence of r-protein operon-encoded genes and genes involved in translation, which are coordinately reduced in expression during early stationary growth. Those genes comprise r-protein genes from most r-protein transcription units including a number of genes co-transcribed within r-protein operons, such as *rimM*, *fusA rpoB *and *rpoC*. Repression affects almost all r-protein operons and as such r-proteins constituting the small and large ribosomal subunits. Moreover, primer extension analysis of the rRNA expression level performed under the same conditions clearly demonstrated a similar reduction, which strongly supports the conclusion that ribosome biogenesis must indeed be inhibited in the 6S RNA deficient strain. In addition to the core components of the ribosome a number of auxiliary genes involved in maintaining an active translation apparatus also show reduced expression. Among those genes are *yibK *and *rimM*, encoding rRNA methylases and *fusA *and *lepA*, encoding translation factors, which further underlines the fact that depletion of 6S RNA results in a coordinated reduction of the components necessary for active translation.

The expression of a number of genes of interest, whether directly or indirectly, is also enhanced during stationary phase. Those genes comprise numerous transcriptional regulators but also genes related to the translation process, such as *smpB*, encoding the protein SmpB that binds to tmRNA and serves to rescue ribosomes translating defective mRNAs [35]. Many genes related to stress signals are found as differentially expressed. One gene, *rssB*, is involved in the degradation of RpoS, the master regulator for stationary phase and stress. RssB is part of a two-component system and as such regulated by phosphorylation. Whether the observed increase in *rssB *mRNA affects RpoS concentration is therefore questionable. A possible functional link between RpoS and 6S RNA is nevertheless an attractive hypothesis but to verify a possible direct connection to this very complex network certainly requires a separate study. In addition, the expression of *folD *is enhanced. This gene encodes a bifunctional 5,10-methylene-tetrahydrofolate dehydrogenase/5,10-methylene-tetrahydrofolate cyclohydrolase. As such it is involved in the one-carbon metabolism and may be considered as functionally related to the gene *ygfA*, a putative 5,10-methenyltetrahydrofolate synthetase, which is co-transcribed with E. coli 6S RNA and found downstream of many enterobacterial and g-proteobacterial ssrS transcription units [7,8]. The functional importance of this co-transcribed protein gene has been elusive, so far. Folate is an important C1-donor in the purine synthesis, however, and considering the observed finding that genes involved in purine metabolism, such as *guaD *and *add *are also enhanced one might speculate that this is the functional link between the two genes *ssrS *and *ygfA*.

### Evidence of a link between 6S RNA and the basal ppGpp concentration in the cell

In contrast to the very high ppGpp levels induced under amino acid deprivation and nutritional stress, which trigger the stringent response [36,37] the basal concentration of ppGpp has been shown to be directly linked to the cellular growth rate and to the adaptation of metabolism during different growth phases [27,38]. In *E. coli *two different enzymes are responsible for the different activities. The ribosome associated RelA protein catalyzes the high ppGpp concentrations in response to the codon-directed binding of a non-aminoacylated tRNA to the ribosomal A-site. On the other hand, the basal ppGpp level mainly results from the balanced synthesis and hydrolysis activities of the bi-functional enzyme SpoT, which acts as growth rate sensor. Changes in the balanced ratio of synthesis and degradation of ppGpp are subject to a variety of cellular compounds and their concentrations, such as carbon sources, iron, phosphate, fatty acids or metabolic compounds linked to stress. All these compounds are potentially acting as growth rate sensors [39]. One could speculate that an altered NTP pool resulting from the differentially expressed enzymes, such as GuaD, FolD or Add may also act as effector for SpoT.

Based on our microarray results and supported by the primer extension and biochemical analysis we propose the following plausible scenario. The major enzyme responsible for adjusting the basal ppGpp levels in *E. coli *is SpoT [27,39]. During stationary growth in the absence of 6S RNA, the delicate balance of the two opposing hydrolytic and synthesizing activities of SpoT responsible to preserve and adjust the basal level of ppGpp, is disturbed, giving rise to an increased level of the effector nucleotide. This increase has no directly measurable impact on the growth rate of the cells but causes a subtle decrease in ribosomal constituents as the major genes, which are directly affected by ppGpp. Note, that genes known to be under positive ppGpp control are also increased in the *ssrS*^- ^strain e.g. *hisL*, *hisH*, *thrL *or several aromatic amino acid transporters (Table 3).

It is rather unlikely that 6S RNA acts as direct effector of SpoT, although uncharged tRNAs have been reported to inhibit the hydrolytic activity of SpoT [40]. If 6S RNA had a similar function one would expect the ppGpp level to drop in the absence of 6S RNA. Considering the direct impact of 6S RNA on the purine metabolism we propose furthermore that an altered level of purine nucleotides is one of the most likely cellular signals, which may affect SpoT activity.

Studies on the transcriptional regulation of 6S RNA indicate that expression of 6S itself is linked to the network known to sense growth changes, e.g. the regulatory proteins H-NS, LRP and FIS [10]. There is no apparent induction of 6S RNA transcription during stationary phase, however, and the observed 6S RNA accumulation is rather due to the metabolic stability of 6S RNA under stationary growth conditions. Generally transcription of 6S RNA is robust and apparently only under conditions of nutritional upshift a notable increase in transcription rate is observed (data not shown). Transcription of 6S RNA is not under stringent control *in vitro *and *in vivo*, when ppGpp reaches millimolar concentrations [7,10]. It is still possible, however, that the 6S RNA promoters respond to the more subtle basal changes in ppGpp concentration as they occur under steady state growth conditions resulting from changes in SpoT activity. Whether or not such an autoregulatory feedback mechanism exists remains to be shown.

## Methods

### Bacterial strains

Strains MC4100 [41] and the isogenic *ssrS*^- ^strain MM139 [18] were a friendly gift from the Beckwith Lab.

### Oligonucleotides used for primer extension

#### Total RNA extraction

Cells were grown in YT-media at 37°C to either log- (~0.6 A_600_) or early stationary phase (~2.4 A_600_). Cells were instantly cooled to 0°C and concentrated by centrifugation. Total RNA was extracted by the hot phenol lysis as described [10]. All RNA samples were digested with RNase-free DNaseI (Roche, Mannheim, Germany). The final RNA concentration was determined by UV spectroscopy and the quality of the preparation was verified by inspection of the integrity of the rRNA fraction using agarose gel electrophoresis.

#### Global gene expression analysis

Cell growth, time points of analysis and preparation of total RNA samples were carried out as described above. Synthesis of Cy3- and Cy5-dUTP labelled cDNA samples were performed as described [42]. The *E. coli *Genome AROS™ V2.0 DNA microarrays were obtained from Operon (Cologne, Germany). The array design includes 9308 longmer oligonucleotide (70 mer) probes, representing genomes of four *E. coli *strains and three plasmids (4269 ORFs in K12, 5306 ORFs in O157:H7, 5251 ORFs in O157:H7, 5366 ORFs in CFT073, 3 genes in OSAK1, 10 genes in pO157_Sakai, 97 genes in pO157_EDL933). Some genes are represented by different gene-specific oligonucleotides (e.g. *guaD*).

Hybridization and stringent washing of the microarrays were performed according to the instructions of the supplier. Hybridization was carried out for 16-18 h at 42°C using a MAUI hybridization system (BioMicro Systems, Salt Lake City, USA). After washing the microarrays were dried by centrifugation (5 min, 1000 × g) and Cy3- and Cy5-fluorescence was determined with 10 μm/pixel resolution using an Axon GenePix 4000B laser scanner (Axon Instruments, USA). Quantitative image analysis was carried out using GenePix image analysis software and results were saved as GPR-file (GenePix Pro 6.0, Axon Instruments). For data normalization, GPR-files were processed using the BioConductor/R-packages limma and marray http://www.bioconductor.org. The processed and loess-normalized data as well as detailed experimental information according to MIAME [43] were stored in the in-house microarray database for further analysis [44].

To assess effects of 6S deletion on global gene expression pattern DNA-microarray analyses of RNA levels of *E. coli ssrS^- ^*mutant MM139 with the wild- type MC4100 were carried out from 2 independent cultures for the exponential time point (2 hybridizations) and from 3 independent cultures for the early stationary phase, which also included a colour-swap (4 hybridizations). To filter for reliable signal detection and differentially expressed genes the following quality filter was applied: (i) flags > = 0 (GenePix Pro 6.0), (ii) signal/noise > = 5 for Cy5 (F635 Median/B635 Median, GenePix Pro 6.0) or Cy3 (F532 Median/B532 Median, GenePix Pro 6.0), (iii) >1.5-fold change in the comparison MM139 versus MC4100. For statistical analysis *p *values were calculated by Student's t-test (Excel, Microsoft).

#### qRT-PCR assay

qRT-PCR analyses were carried out with the Mastercycler ep realplex system (Eppendorf) and its instrument software (version 1.0) using the QuantiTect SYBR Green RT-PCR Kit (Qiagen). To generate single-stranded cDNA, 2 μg of isolated total RNA were reverse transcribed with Superscript II (Invitrogen), using random hexamer primers. To generate no amplification control (NAC) from total RNA, the enzyme Superscript II was omitted in separate reactions. The primer pairs for realtime PCR (170-176 bp product size) of *osmY *(5'-TCGATGACTATGACAAGACTGAAGA, 5'-GCTGTCATCCATGAAATTACC), *rpoD *(5'-CAAACAGTTCCGCCTGGT, 5'-AGGTATCGCTGGTTTCGTTG), *bolA *(5'-TGGTCAGCGATCGTTTTACG, 5'-GACAGGGAGGAGAGGCAAAG) and *rrsA *as a control (5'-GTAATACGGAGGGTGCAAGC, 5'-TACGCATTTCACCGCTACAC) were designed based on gene sequences using web-based Primer3 http://frodo.wi.mit.edu/primer3/. Primer pairs were checked for PCR amplification and specificity using *E. coli *genomic DNA as template. To test for contaminations in RNA samples with genomic DNA, the integrity and purity of generated cDNA and NAC were checked by qualitative PCR before real-time quantification. The cDNA template used for DNA amplification was equivalent to 10 ng RNA per 50 μl qRT-PCR reaction. The qRT-PCR reactions were performed in duplicate for each gene together with 1 reaction of NTC and NAC. After the qRT-PCR reaction PCR products were further characterized by melting curve analysis. The threshold cycles (C_T_) were calculated by the instruments software. The relative gene expression levels were calculated by comparing the C_T _values using REST 2005 software [45].

#### Quantitative primer extension

Quantitative primer extension was performed as described [10]. For mRNA analysis 5 μg total RNA was incubated with 0.5 pmol specific oligonucleotide primer, which had been labelled with γ^32^P-ATP (Hartmann Analytics, Braunschweig, Germany) and T4 polynucleotide kinase (NEB). Samples were heated for 3 minutes to 70°C and hybridisation was performed by slow cooling to room temperature. Primer extension reaction was stopped after 30 minutes at 42°C adding 5 μl formamide loading buffer. The samples were then denatured at 100°C for 5 minutes and separated on 15% polyacrylamide gels.

#### Quantitative assessment of basal ppGpp levels

Determination of basal ppGpp concentrations *in vivo *was performed as described [46]. Cells were grown under same conditions and time points as for the microarray analysis. A 100 μl aliquot of the cell culture was labelled with 20 μCi ^32^P neutralized phosphoric acid for 1 h and 40 μl of this cell suspension were directly mixed with 1N formic acid and frozen immediately in liquid nitrogen. Cells were disrupted by three cycles of freeze-thawing, followed by 10 minutes centrifugation at 13000 rpm. The supernatant subsequently used for thin layer chromatography. As positive control high ppGpp concentrations were induced by the stringent control. In this case MG1655 cells were grown in YT-media to mid-exponential phase (OD_600 _0.6), labelled for 15 minutes with ^32^P neutralized phosphoric acid, followed by the addition of 0.7 mg/ml serine hydroxamate [47]. The supernatant was then used for thin layer chromatography.

## Authors' contributions

TN and RG carried out the molecular genetic studies. TP carried out the microarray analysis and data evaluation. RW conceived of the study, participated in its design and coordination and wrote the manuscript. All authors read and approved the final manuscript.

## Supplementary Material

Additional file 1 **Promoter specificities of genes analyzed by primer extension**. The table indicates different promoter specificities of selected genes analyzed by primer extension. Click here for file

Additional file 2 **Growth curve of wild-type (MM139) and *ssrS*^-^(MC4100) strains**. The figure shows the growth curves of wild-type and mutant strains and the time points of RNA extraction. Click here for file

Additional file 3 **Differentially expressed genes during exponential growth**. The table lists all genes >1.5-fold differentially expressed in the DNA microarray analysis comparing the *ssrS*^- ^strain MM139 with the wild type MC4100 during exponential growth. Click here for file

Additional file 4 **Differentially expressed genes during stationary growth**. The table lists all genes >1.5-fold differentially expressed in the DNA microarray analysis comparing the *ssrS*^- ^strain MM139 with the wild type MC4100 during stationary growth. Click here for file

Additional file 5 **Comparison of selected miroarray data with qRT-PCR analysis**. The table lists a comparison of DNA-microarray data and qRT-PCR analysis for selected genes in early stationary phase. Click here for file
