# Supplementary material for: Depletion of the non-coding regulatory 6S RNA in E. coli causes a surprising reduction in the expression of the translation machinery
Source: BMC Genomics. 2010 Mar 11;11:165. doi: 10.1186/1471-2164-11-165 (PMC2848244; doi:10.1186/1471-2164-11-165)
Supplement: Additional file 1 — Promoter specificities of genes analyzed by primer extension. The table indicates different promoter specificities of selected genes analyzed by primer extension. [file 1471-2164-11-165-S1.DOC]

## Additional file 1

## Table S1: Promoter specificities of genes analyzed by primer extension

| Gene | **Promoter/**  **specificity** | **Characteristics** | **Reference** |
| --- | --- | --- | --- |
| *bolA* | P1  σ^38^ > σ^70^-dependent | stationary phase expression | Nguyen and Burgess, 1997  Aldea et al., 1989 |
| *osmY* | P  σ38/σ^70^-dependent | stationary phase expression | Lange et al., 1993 |
| *fic* | P  σ^38^-dependent | stationary phase expression | Hiratsu et al., 1995 |
| *rpoD* | P3  σ^70^-dependent^1)^ | expression throughout the growth curve | Lupsky et al., 1984 |
| *rrn* | P1  σ^70^-dependent | exponential phase expression | Wagner, 2000 |

^1)^ Transcription from *rpoD* P3 has originally been assigned as σ^32^-dependent [48] but this promoter shows all σ^70^-specific characteristics
