# Supplementary material for: Depletion of the non-coding regulatory 6S RNA in E. coli causes a surprising reduction in the expression of the translation machinery
Source: BMC Genomics. 2010 Mar 11;11:165. doi: 10.1186/1471-2164-11-165 (PMC2848244; doi:10.1186/1471-2164-11-165)
Supplement: Additional file 2 — Growth curve of wild-type (MM139) and ssrS-(MC4100) strains. The figure shows the growth curves of wild-type and mutant strains and the time points of RNA extraction. [file 1471-2164-11-165-S2.DOC]

**Additional file 2**

**
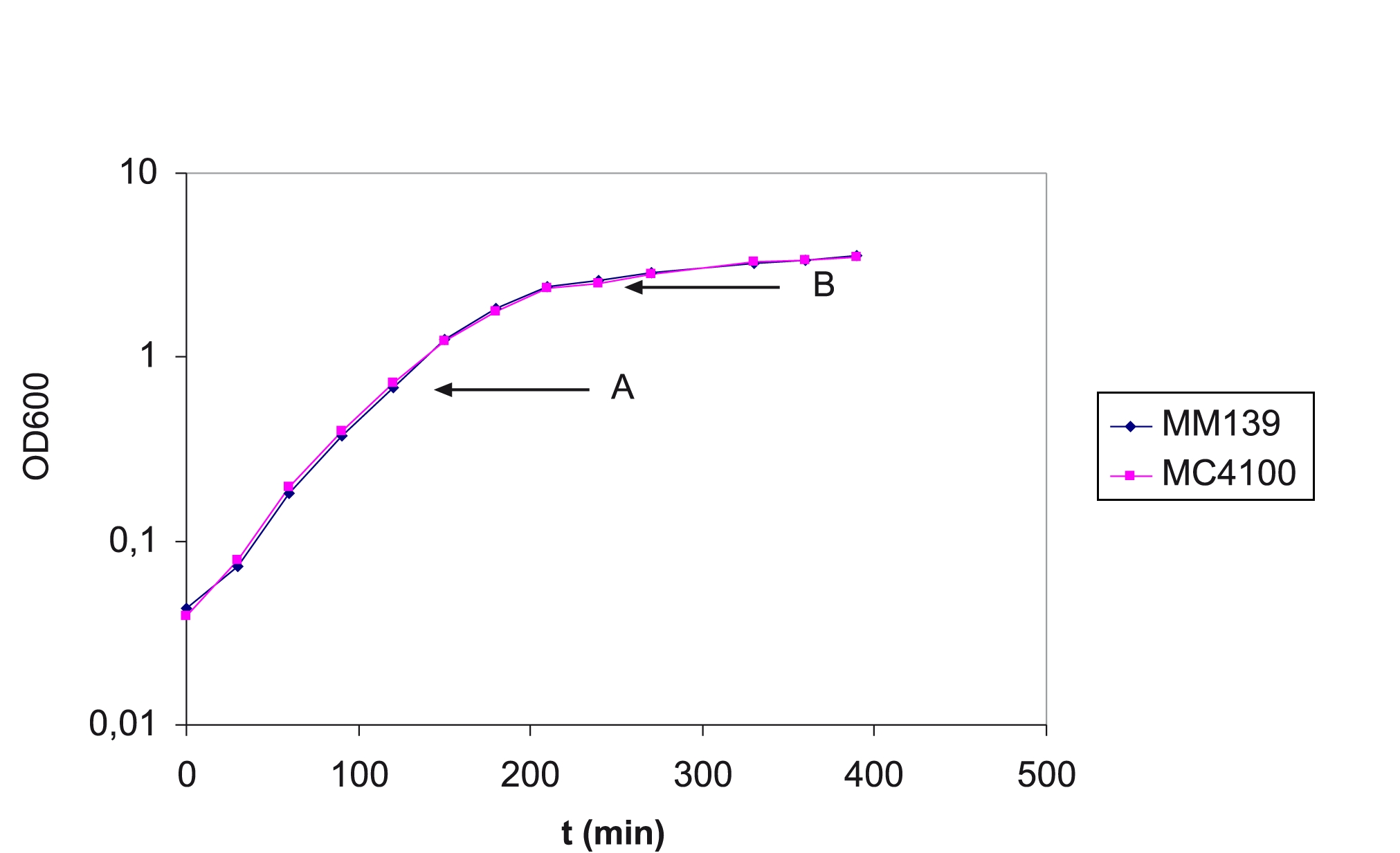
**

**Figure S1: Growth curve of wild-type (MM139) and *ssrS*^-^ (MC4100) strains**

Cells were grown in YT-media at 37°C and at the indicated times (A, for exponential growth, B, for early stationary growth) samples were withdrawn for total RNA extraction.
