# Supplementary material for: Depletion of the non-coding regulatory 6S RNA in E. coli causes a surprising reduction in the expression of the translation machinery
Source: BMC Genomics. 2010 Mar 11;11:165. doi: 10.1186/1471-2164-11-165 (PMC2848244; doi:10.1186/1471-2164-11-165)
Supplement: Additional file 3 — Differentially expressed genes during exponential growth. The table lists all genes >1.5-fold differentially expressed in the DNA microarray analysis comparing the ssrS- strain MM139 with the wild type MC4100 during exponential growth. [file 1471-2164-11-165-S3.DOC]

**Table S2:**  Genes >1.5-fold differentially expressed in the DNA microarray analysis comparing the transcriptome of the *ssrS^-^* strain MM139 with the wild type MC4100 in the exponential growth phase. The relative mRNA values represent mean of two biological replicates.

| AROS™ V2.0 K12 DNA Oligo | b# | Gene | mRNA level *ssrS*^-^ / Wt | p-value | Annotation | Promotors according to Regulon DB [33] |
| --- | --- | --- | --- | --- | --- | --- |
| E100002819 | *b2883* | *guaD* | 18.41 | 0.0612 | guanine deaminase |  |
| E100002821 | *b4464* | *ygfQ* | 12.78 | 0.0322 | predicted transporter |  |
| E200002177 | *b2883* | *guaD* | 11.76 | 0.0117 | guanine deaminase |  |
| E100002820 | *b4464* | *ygfQ* | 10.84 | 0.0080 | predicted transporter |  |
| E100003050 | *b3117* | *tdcB* | 4.69 | 0.1876 | threonine dehydratase | unknown |
| E100003051 | *b3118* | *tdcA* | 4.23 | 0.2080 | DNA-binding transcriptional activator | unknown |
| E100003049 | *b3116* | *tdcC* | 3.48 | 0.1802 | L-threonine/L-serine transporter |  |
| E100000774 | *b0798* | *ybiA* | 2.86 | 0.1145 | hypothetical protein |  |
| E100001567 | *b1597* | *asr* | 2.53 | 0.0503 | acid shock protein precursor | σ^38^ |
| E100000788 | *b0812* | *dps* | 2.34 | 0.1535 | DNA protection during starvation conditions | σ^38^,  σ^70^ |
| E100003059 | *b3127* | *garP* | 2.27 | 0.0530 | predicted (D)-galactarate transporter | unknown |
| E100004008 | *b4116* | *adiY* | 2.26 | 0.1441 | DNA-binding transcriptional activator | unknown |
| E100003058 | *b3126* | *garL* | 2.19 | 0.1276 | alpha-dehydro-beta-deoxy-D-glucarate aldolase |  |
| E100003419 | *b3496* | *yhiP* | 2.12 | 0.1269 | predicted transporter |  |
| E100001593 | *b1623* | *add* | 2.09 | 0.1214 | adenosine deaminase |  |
| E100003048 | *b3115* | *tdcD* | 2.03 | 0.1489 | acetate/propionate kinase |  |
| E100002729 | *b2789* | *gudP* | 2.03 | 0.0962 | predicted D-glucarate transporter | unknown |
| E100000690 | *b0707* | *ybgA* | 2.03 | 0.0469 | hypothetical protein | σ^38^ |
| E100000824 | *b0848* | *ybjM* | 2.02 | 0.2107 | predicted inner membrane protein |  |
| E100000767 | *b0791* | *ybhQ* | 2.01 | 0.1038 | predicted inner membrane protein | unknown |
| E100002741 | *b2801* | *fucP* | 1.99 | 0.0884 | L-fucose transporter | σ^70^ |
| E100000716 | *b0733* | *cydA* | 1.97 | 0.2125 | cytochrome d terminal oxidase, subunit I | σ^70^ |
| E100000018 | *b0018* | *mokC* | 1.97 | 0.0498 | regulatory protein for HokC, overlaps CDS of hokC |  |
| E200000315 | *b1480* | *sra* | 1.97 | 0.1333 | 30S ribosomal subunit protein S22 | σ^38^ |
| E200002122 | *b2789* | *gudP* | 1.96 | 0.0699 | predicted D-glucarate transporter | unknown |
| E100001451 | *b1480* | *sra* | 1.94 | 0.1635 | 30S ribosomal subunit protein S22 | σ^38^ |
| E100002642 | *b2702* | *srlA* | 1.93 | 0.0998 | glucitol/sorbitol-specific enzyme IIC component of PTS | σ^70^ |
| E100001693 | *b1725* | *yniA* | 1.93 | 0.1101 | predicted phosphotransferase/kinase |  |
| E100005859 | *b4457* | *csrC* | 1.93 | 0.0835 | regulatory RNA | unknown |
| E100003997 | *b4105* | *phnD* | 1.92 | 0.2756 | phosphonate/organophosphate ester transporter subunit |  |
| E100002812 | *b2876* | *yqeC* | 1.92 | 0.0898 | hypothetical protein |  |
| E200000322 | *b1426* | *ydcH* | 1.90 | 0.1070 | hypothetical protein |  |
| E100001707 | *b1739* | *osmE* | 1.90 | 0.0740 | DNA-binding transcriptional activator | σ^38^,  σ^70^ |
| E100003047 | *b3114* | *tdcE* | 1.89 | 0.0142 | pyruvate formate-lyase 4/2-ketobutyrate formate-lyase |  |
| E100002301 | *b2343* | *yfcZ* | 1.89 | 0.0299 | hypothetical protein |  |
| E100002615 | *b2670* | *ygaW* | 1.88 | 0.1468 | predicted inner membrane protein |  |
| E100001397 | *b1426* | *ydcH* | 1.87 | 0.0521 | hypothetical protein |  |
| E100002544 | *b2597* | *yfiA* | 1.85 | 0.1541 | cold shock protein associated with 30S ribosomal subunit | σ^32^ |
| E100003060 | *b3128* | *garD* | 1.85 | 0.1333 | (D)-galactarate dehydrogenase | unknown |
| E200000118 | *b2426* | *ucpA* | 1.82 | 0.0744 | short chain dehydrogenase | unknown |
| E200000887 | *b0791* | *ybhQ* | 1.81 | 0.0467 | predicted inner membrane protein | unknown |
| E100003020 | *b3087* | *ygjR* | 1.80 | 0.1028 | predicted NAD(P)-binding dehydrogenase |  |
| E100001923 | *b1959* | *yedA* | 1.80 | 0.0794 | predicted inner membrane protein |  |
| E100001788 | *b1820* | *yobD* | 1.80 | 0.0962 | hypothetical protein |  |
| E100004040 | *b4149* | *blc* | 1.79 | 0.0195 | outer membrane lipoprotein (lipocalin) | σ^38^ |
| E100002784 | *b2847* | *yqeI* | 1.78 | 0.2323 | predicted transcriptional regulator |  |
| E100003288 | *b3365* | *nirB* | 1.77 | 0.2155 | nitrite reductase, large subunit, NAD(P)H-binding | σ^70^ |
| E100000129 | *b0129* | *yadI* | 1.77 | <0.0001 | predicted PTS Enzyme IIA |  |
| E100004111 | *b4223* | *yzfA* | 1.76 | 0.2778 | hypothetical protein |  |
| E100003681 | *b3766* | *ilvL* | 1.76 | 0.0740 | ilvG operon leader peptide | σ^70^ |
| E200000850 | *b0699* | *ybfA* | 1.75 | 0.1814 | hypothetical protein |  |
| E200002421 | *b3126* | *garL* | 1.74 | 0.1155 | alpha-dehydro-beta-deoxy-D-glucarate aldolase |  |
| E200002431 | *b3155* | *yhbQ* | 1.71 | 0.0974 | hypothetical protein |  |
| E100001792 | *b1824* | *yobF* | 1.71 | 0.1048 | hypothetical protein |  |
| E200000013 | *b1420 b4428* | *mokB hokB* | 1.71 | 0.1255 | regulatory peptide | "toxic polypeptide, small " |  |
| E100003284 | *b3361* | *fic* | 1.71 | 0.0885 | stationary-phase protein, cell division | σ^38^ |
| E200000169 | *b4515* | *ybgT* | 1.70 | 0.1974 | hypothetical protein |  |
| E100003087 | *b3155* | *yhbQ* | 1.69 | 0.1080 | hypothetical protein |  |
| E100002647 | *b2707* | *srlR* | 1.69 | 0.1327 | DNA-bindng transcriptional repressor | unknown |
| E100002101 | *b2142* | *yohK* | 1.68 | 0.1699 | predicted inner membrane protein |  |
| E100002813 | *b2877* | *ygfJ* | 1.68 | 0.1310 | hypothetical protein |  |
| E100002770 | *b2833* | *ygdR* | 1.68 | 0.0920 | hypothetical protein |  |
| E100003832 | *b3928* | *yiiU* | 1.68 | 0.0958 | hypothetical protein |  |
| E100002892 | *b2957* | *ansB* | 1.68 | 0.2417 | periplasmic L-asparaginase II | σ^70^, unknown |
| E200000157 | *b4189* | *yjfO* | 1.68 | 0.0392 | hypothetical protein |  |
| E100001965 | *b2006* | *yeeW* | 1.67 | 0.1935 | CP4-44 prophage; predicted protein |  |
| E100002166 | *b2208* | *napF* | 1.67 | 0.1544 | ferredoxin-type protein, predicted role in electron transfer to periplasmic nitrate reductase (NapA) | σ^70^, unknown |
| E100003477 | *b3555* | *yiaG* | 1.67 | 0.0302 | predicted transcriptional regulator | σ^38^ |
| E100000717 | *b0734* | *cydB* | 1.66 | 0.2060 | cytochrome d terminal oxidase, subunit II |  |
| E200000266 | *b4576* | *insB-7* | 1.66 | 0.1845 | - |  |
| E100003478 | *b3556* | *cspA* | 1.66 | 0.0388 | major cold shock protein | σ^70^ |
| E100002811 | *b2875* | *yqeB* | 1.66 | 0.0861 | conserved protein with NAD(P)-binding Rossman fold |  |
| E100002728 | *b2788* | *gudX* | 1.63 | 0.1465 | predicted glucarate dehydratase |  |
| E200001968 | *b4440* | *ryfA* | 1.62 | 0.1825 | unknown RNA |  |
| E200000145 | *b3646* | *yicG* | 1.61 | 0.1749 | conserved inner membrane protein |  |
| E100003628 | *b3707* | *tnaC* | 1.61 | 0.0145 | tryptophanase leader peptide | σ^70^ |
| E100003833 | *b3929* | *menG* | 1.61 | 0.0582 | ribonuclease activity regulator protein RraA | σ^38^ |
| E100002617 | *b2672* | *ygaM* | 1.60 | 0.0752 | hypothetical protein |  |
| E200000115 | *b2378* | *ddg* | 1.60 | 0.1065 | lipid A biosynthesis palmitoleoyl acyltransferase | σ^24^ |
| E100003595 | *b3674* | *yidF* | 1.60 | 0.0624 | predicted DNA-binding transcriptional regulator |  |
| E100000162 | *b0162* | *cdaR* | 1.60 | 0.1397 | DNA-binding transcriptional activator | unknown |
| E100004114 | *b4226* | *ppa* | 1.60 | 0.1077 | inorganic pyrophosphatase | unknown |
| E100000894 | *b0919* | *ycbJ* | 1.60 | 0.2249 | hypothetical protein |  |
| E100000641 | *b0651* | *rihA* | 1.59 | 0.1069 | ribonucleoside hydrolase 1 |  |
| E200000547 | *b0146* | *sfsA* | 1.59 | 0.1130 | sugar fermentation stimulation protein A | σ^70^ |
| E100003687 | *b3773* | *ilvY* | 1.59 | 0.0717 | DNA-binding transcriptional dual regulator | σ^70^ |
| E200000149 | *b3885* | *yihX* | 1.59 | 0.1844 | phosphatase |  |
| E200000178 | *b4529* | *ydbJ* | 1.59 | 0.1202 | hypothetical protein |  |
| E200002400 | *b3074* | *ygjH* | 1.59 | 0.1782 | hypothetical protein |  |
| E100002975 | *b3041* | *ribB* | 1.59 | 0.0595 | 3,4-dihydroxy-2-butanone 4-phosphate synthase |  |
| E100001630 | *b1660* | *ydhC* | 1.58 | 0.1245 | predicted transporter |  |
| E100000597 | *b0607* | *uspG* | 1.58 | 0.1320 | universal stress protein UP12 | unknown |
| E100003927 | *b4035* | *malK* | 1.58 | 0.0266 | fused maltose transport subunit, ATP-binding | σ^70^ |
| E100002263 | *b2305* | *yfcI* | 1.57 | 0.0939 | hypothetical protein |  |
| E100000738 | *b0762* | *ybhT* | 1.57 | 0.1897 | hypothetical protein |  |
| E200001389 | *b1376* | *uspF* | 1.57 | 0.1451 | stress-induced protein, ATP-binding protein | unknown |
| E100005853 | *b4558* | *yifL* | 1.56 | 0.1264 | predicted lipoprotein | σ^70^ |
| E100001160 | *b1187* | *fadR* | 1.56 | 0.1826 | fatty acid metabolism regulator | unknown |
| E100000633 | *b0643* | *ybeL* | 1.56 | 0.0408 | hypothetical protein | unknown |
| E100003292 | *b3369* | *yhfL* | 1.56 | 0.2453 | conserved secreted peptide |  |
| E100001178 | *b1205* | *ychH* | 1.56 | 0.1977 | predicted inner membrane protein | σ^70^ |
| E100000451 | *b0460* | *hha* | 1.56 | 0.2106 | modulator of gene expression, with H-NS |  |
| E100001191 | *b1218* | *chaC* | 1.55 | 0.2285 | regulatory protein for cation transport | σ54 |
| E100004285 | *b4401* | *arcA* | 1.55 | 0.0299 | DNA-binding response regulator in two-component regulatory system with ArcB or CpxA | σ^70^ |
| E100000266 | *b0274, b0264, b0021, b1893, b3445, b0988* |  | 1.55 | 0.1815 | CP4-6 prophage; IS1 transposase InsAB' | CP4-6 prophage; IS1 transposase InsAB' | IS1 transposase InsAB' | IS1 transposase InsAB' | IS1 transposase InsAB' | IS1 transposase InsAB' | σ^24^,  σ^70^, unknown |
| E200000133 | *b2964* | *nupG* | 1.55 | 0.1056 | nucleoside transporter | σ^70^ |
| E100001968 | *b2009* | *sbmC* | 1.54 | 0.1250 | DNA gyrase inhibitor | unknown |
| E200002413 | *b3103* | *yhaH* | 1.54 | 0.1299 | predicted inner membrane protein |  |
| E200000026 | *b0434* | *yajG* | 1.54 | 0.1719 | predicted lipoprotein |  |
| E100000475 | *b0484* | *copA* | 1.54 | 0.2575 | copper transporter | unknown |
| E100001033 | *b1060* | *yceP* | 1.54 | 0.0991 | hypothetical protein | σ^32^, unknown |
| E100003947 | *b4055* | *aphA* | 1.53 | 0.1443 | acid phosphatase/phosphotransferase, class B, non-specific |  |
| E100001279 | *b1308* | *pspE* | 1.53 | 0.1207 | thiosulfate:cyanide sulfurtransferase rhodanese) | unknown |
| E100002095 | *b2136* | *yohD* | 1.53 | 0.0773 | conserved inner membrane protein |  |
| E100003937 | *b4045* | *yjbJ* | 1.53 | 0.1464 | predicted stress response protein |  |
| E100002805 | *b2869* | *ygeV* | 1.53 | 0.1377 | predicted DNA-binding transcriptional regulator |  |
| E100004018 | *b4126* | *yjdI* | 1.52 | 0.0987 | hypothetical protein |  |
| E100003259 | *b3336* | *bfr* | 1.52 | 0.1196 | bacterioferritin, iron storage and detoxification protein |  |
| E100000030 | *b0030* | *rihC* | 1.52 | 0.0188 | ribonucleoside hydrolase 3 |  |
| E200000974 | *b0887* | *cydD* | 1.52 | 0.0353 | fused cysteine transporter subunits of ABC | unknown |
| E100000110 | *b0110* | *ampD* | 1.52 | 0.0391 | N-acetyl-anhydromuranmyl-L-alanine amidase |  |
| E100004019 | *b4127* | *yjdJ* | 1.52 | 0.1106 | predicted acyltransferase with acyl-CoA N-acyltransferase domain |  |
| E100003447 | *b3524* | *yhjG* | 1.52 | 0.0306 | predicted outer membrane biogenesis protein |  |
| E100002833 | *b2897* | *ygfY* | 1.52 | 0.0540 | hypothetical protein |  |
| E100003831 | *b3927* | *glpF* | 1.50 | 0.1686 | glycerol facilitator | σ^70^ |
| E100002707 | *b2767* | *ygcO* | 1.50 | 0.1472 | predicted 4Fe-4S cluster-containing protein |  |
| E100003333 | *b3410* | *yhgG* | 1.50 | 0.2398 | predicted DNA-binding transcriptional regulator |  |
| E200000101 | *b2007* | *yeeX* | 1.50 | 0.2070 | hypothetical protein |  |
| E200001999 | *b2617* | *smpA* | 1.50 | 0.1781 | small membrane lipoprotein | σ^24^ |
| E100003655 | *b3734* | *atpA* | 0.66 | 0.2187 | F0F1 ATP synthase subunit alpha |  |
| E100002052 | *b2093* | *gatB* | 0.66 | 0.0179 | galactitol-specific enzyme IIB component of PTS |  |
| E100000770 | *b0794* | *ybhF* | 0.66 | 0.2291 | fused predicted transporter subunits of ABC superfamily: ATP-binding components |  |
| E100001376 | *b4492* | *ydbA* | 0.66 | 0.0125 | - |  |
| E100002781 | *b2844* | *yqeF* | 0.66 | 0.2591 | acetyl-CoA acetyltransferase |  |
| E100001423 | *b1452* | *yncE* | 0.66 | 0.1620 | hypothetical protein |  |
| E100000251 | *b0656, b0552, b3505, b3218, b0259, b2192, b2030, b4571, b1994, b2982, b1331, b1370* |  | 0.66 | 0.1639 | IS5 transposase and trans-activator | DLP12 prophage; IS5 transposase and trans-activator | IS5 transposase and trans-activator | IS5 transposase and trans-activator | CP4-6 prophage; IS5 transposase and trans-activator | IS5 transposase and trans-ac tivator | σ^24^,  σ^70^, unknown |
| E100002481 | *b2530* | *iscS* | 0.66 | 0.1571 | cysteine desulfurase |  |
| E100001932 | *b1968* | *yedV* | 0.66 | 0.0867 | predicted sensory kinase in two-component regulatory system with YedW |  |
| E100001109 | *b1136* | *icdA* | 0.66 | 0.0438 | isocitrate dehydrogenase |  |
| E100002245 | *b2287* | *nuoB* | 0.66 | 0.1532 | NADH dehydrogenase subunit B |  |
| E100001794 | *b1826* | *yobG* | 0.66 | 0.0700 | hypothetical protein | unknown |
| E100000969 | *b0995* | *torR* | 0.66 | 0.1693 | DNA-binding response regulator in two-component regulatory system with TorS | σ^70^ |
| E100004096 | *b4208* | *cycA* | 0.66 | 0.2888 | D-alanine/D-serine/glycine transporter | unknown |
| E200000046 | *b1562* | *hokD* | 0.66 | 0.2819 | Qin prophage; small toxic polypeptide |  |
| E100002968 | *b3034* | *nudF* | 0.66 | 0.2745 | ADP-ribose pyrophosphatase |  |
| E100002862 | *b2927* | *epd* | 0.66 | 0.0425 | D-erythrose 4-phosphate dehydrogenase | σ^70^ |
| E100004166 | *b4279* | *yjhB* | 0.66 | 0.0800 | KpLE2 phage-like element; predicted transporter |  |
| E100003907 | *b4015* | *aceA* | 0.65 | 0.0058 | isocitrate lyase |  |
| E100003656 | *b3735* | *atpH* | 0.65 | 0.2311 | F0F1 ATP synthase subunit delta |  |
| E100001077 | *b1104* | *ycfL* | 0.65 | 0.1468 | hypothetical protein |  |
| E100003869 | *b3965* | *trmA* | 0.65 | 0.0784 | tRNA (uracil-5-)-methyltransferase | σ^32^ |
| E100000989 | *b1015* | *putP* | 0.65 | 0.2409 | proline:sodium symporter | σ^70^, unknown |
| E100001446 | *b1475* | *fdnH* | 0.65 | 0.1809 | formate dehydrogenase-N, Fe-S (beta) subunit, nitrate-inducible |  |
| E100001991 | *b2032* | *wbbK* | 0.65 | 0.0165 | lipopolysaccharide biosynthesis protein |  |
| E100001954 | *b4582* | *yoeA* | 0.65 | 0.0002 | - |  |
| E100002554 | *b2607* | *trmD* | 0.65 | 0.1400 | tRNA (guanine-N(1)-)-methyltransferase |  |
| E100000673 | *b0690* | *ybfG* | 0.65 | 0.2083 | hypothetical protein |  |
| E100001063 | *b1090* | *plsX* | 0.65 | 0.1839 | fatty acid/phospholipid synthesis protein |  |
| E100001652 | *b1684* | *sufA* | 0.65 | 0.1610 | iron-sulfur cluster assembly scaffold protein | σ^70^ |
| E100003666 | *b3745* | *yieM* | 0.65 | 0.2787 | predicted von Willebrand factor containing protein |  |
| E100002747 | *b2807* | *ygdD* | 0.65 | 0.1526 | conserved inner membrane protein |  |
| E100003658 | *b3737* | *atpE* | 0.64 | 0.0694 | F0F1 ATP synthase subunit C |  |
| E100002246 | *b2288* | *nuoA* | 0.64 | 0.1794 | NADH dehydrogenase subunit A | σ^70^, unknown |
| E100002270 | *b2312* | *purF* | 0.64 | 0.1073 | amidophosphoribosyltransferase |  |
| E100001247 | *b1276* | *acnA* | 0.64 | 0.1497 | aconitate hydratase | σ^38^,  σ^70^, unknown |
| E100002748 | *b2808* | *gcvA* | 0.64 | 0.1463 | DNA-binding transcriptional dual regulator | σ^70^ |
| E100001690 | *b1722* | *ydiY* | 0.64 | 0.2455 | hypothetical protein |  |
| E200001875 | *b2309* | *hisJ* | 0.63 | 0.1502 | histidine/lysine/arginine/ornithine transporter subunit | σ^70^ |
| E100003557 | *b3635* | *mutM* | 0.63 | 0.2758 | formamidopyrimidine-DNA glycosylase | σ^32^ |
| E100002174 | *b2216* | *rcsD* | 0.63 | 0.0155 | phosphotransfer intermediate protein in two-component regulatory system with RcsBC |  |
| E100002914 | *b2980* | *glcC* | 0.63 | 0.2428 | DNA-binding transcriptional dual regulator, glycolate-binding | σ^70^ |
| E200000965 | *b0868* | *ybjS* | 0.63 | 0.2211 | predicted NAD(P)H-binding oxidoreductase with NAD(P)-binding Rossmann-fold domain |  |
| E100001410 | *b1439* | *ydcR* | 0.63 | 0.0871 | fused predicted DNA-binding transcriptional regulator |  |
| E100003242 | *b3319* | *rplD* | 0.63 | 0.2250 | 50S ribosomal protein L4 |  |
| E100000424 | *b0432* | *cyoA* | 0.62 | 0.1646 | cytochrome o ubiquinol oxidase subunit II | σ^70^ |
| E100002150 | *b0656, b0552, b3505, b3218, b0259, b2192, b2030, b4571, b1994, b2982, b1331, b1370* |  | 0.62 | 0.1036 | IS5 transposase and trans-activator | DLP12 prophage; IS5 transposase and trans-activator | IS5 transposase and trans-activator | IS5 transposase and trans-activator | CP4-6 prophage; IS5 transposase and trans-activator | IS5 transposase and trans-activator | σ^24^,  σ^70^, unknown |
| E100001097 | *b1124* | *potC* | 0.62 | 0.1906 | spermidine/putrescine ABC transporter membrane protein |  |
| E100002689 | *b2749* | *ygbE* | 0.62 | 0.1734 | conserved inner membrane protein |  |
| E100001497 | *b1526* | *yneJ* | 0.62 | 0.1616 | predicted DNA-binding transcriptional regulator |  |
| E100002108 | *b2149* | *mglA* | 0.62 | 0.3086 | fused methyl-galactoside transporter subunits of ABC superfamily: ATP-binding components | unknown |
| E100001875 | *b1907* | *tyrP* | 0.62 | 0.1704 | tyrosine transporter | σ^70^, unknown |
| E100002450 | *b2499* | *purM* | 0.62 | 0.2211 | phosphoribosylaminoimidazole synthetase | σ^70^ |
| E200002803 | *b3728* | *pstS* | 0.62 | 0.1127 | phosphate transporter subunit | σ^38^,  σ^70^ |
| E100003526 | *b3604* | *lldR* | 0.62 | 0.2538 | DNA-binding transcriptional repressor |  |
| E100002168 | *b2210* | *mqo* | 0.61 | 0.1877 | malate:quinone oxidoreductase |  |
| E100000855 | *b0879* | *macB* | 0.61 | 0.0596 | fused macrolide transporter subunits of ABC | σ^32^ |
| E100000710 | *b0727* | *sucB* | 0.61 | 0.2196 | dihydrolipoamide acetyltransferase |  |
| E100003153 | *b3223* | *nanE* | 0.61 | 0.2245 | predicted N-acetylmannosamine-6-P epimerase |  |
| E100003514 | *b3592* | *yibF* | 0.61 | 0.0977 | predicted glutathione S-transferase |  |
| E200000060 | *b4510, b0556, b4528, b1362* | *rzoD,*  *rzpD,*  *rzoR, rzpR* | 0.61 | 0.0021 | DLP12 prophage; predicted lipoprotein | DLP12 prophage; predicted murein endopeptidase | Rac prophage; predicted lipoprotein | Rac prophage; predicted defective peptidase | σ^24^,  σ^70^, unknown |
| E100000759 | *b0783* | *moaC* | 0.61 | 0.0865 | molybdenum cofactor biosynthesis protein C |  |
| E200000804 | *b0592* | *fepB* | 0.61 | 0.2367 | iron-enterobactin transporter subunit | σ^70^ |
| E100001291 | *b1320* | *ycjW* | 0.61 | 0.0795 | predicted DNA-binding transcriptional regulator |  |
| E100000513 | *b0522* | *purK* | 0.61 | 0.3188 | phosphoribosylaminoimidazole carboxylase |  |
| E100000709 | *b0726* | *sucA* | 0.61 | 0.1852 | alpha-ketoglutarate decarboxylase | σ^38^ |
| E100002271 | *b2313* | *cvpA* | 0.61 | 0.1363 | membrane protein required for colicin V production | σ^70^, unknown |
| E100000758 | *b0782* | *moaB* | 0.60 | 0.0117 | molybdopterin biosynthesis protein B | unknown |
| E100002616 | *b2671* | *ygaC* | 0.60 | 0.2077 | hypothetical protein |  |
| E100002538 | *b2587* | *kgtP* | 0.60 | 0.2416 | alpha-ketoglutarate transporter |  |
| E200002533 | *b3425* | *glpE* | 0.60 | 0.1775 | thiosulfate sulfurtransferase | unknown |
| E100004146 | *b4258* | *valS* | 0.60 | 0.1595 | valyl-tRNA synthetase | σ^70^ |
| E100002256 | *b2298* | *yfcC* | 0.60 | 0.0169 | predicted inner membrane protein |  |
| E100001299 | *b1328* | *ycjZ* | 0.60 | 0.0557 | predicted DNA-binding transcriptional regulator |  |
| E200000237 | *b0784* | *moaD* | 0.60 | 0.1431 | molybdopterin synthase, small subunit |  |
| E200001386 | *b1344* | *ydaO* | 0.59 | 0.2165 | predicted C32 tRNA thiolase |  |
| E100003274 | *b3351* | *yheR* | 0.58 | 0.2237 | glutathione-regulated potassium-efflux system ancillary protein |  |
| E100001650 | *b1682* | *sufC* | 0.58 | 0.1493 | cysteine desulfurase ATPase component |  |
| E200001872 | *b2306* | *hisP* | 0.58 | 0.2031 | histidine/lysine/arginine/ornithine transporter subunit |  |
| E100000706 | *b0723* | *sdhA* | 0.58 | 0.2070 | succinate dehydrogenase flavoprotein subunit |  |
| E100003649 | *b3728* | *pstS* | 0.58 | 0.1304 | phosphate transporter subunit | σ^38^,  σ^70^ |
| E100000920 | *b0945* | *pyrD* | 0.57 | 0.0443 | dihydroorotate dehydrogenase | σ^70^ |
| E100000068 | *b0068* | *tbpA* | 0.57 | 0.1729 | thiamin transporter subunit |  |
| E200002659 | *b3604* | *lldR* | 0.57 | 0.2505 | DNA-binding transcriptional repressor |  |
| E100001035 | *b1062* | *pyrC* | 0.57 | 0.2149 | dihydroorotase | σ^70^ |
| E100003348 | *b3425* | *glpE* | 0.56 | 0.1944 | thiosulfate sulfurtransferase | unknown |
| E100001162 | *b1189* | *dadA* | 0.56 | 0.1925 | D-amino acid dehydrogenase small subunit | σ^70^ |
| E100002266 | *b2308* | *hisQ* | 0.56 | 0.1622 | histidine/lysine/arginine/ornithine transporter subunit |  |
| E100000850 | *b0874* | *ybjE* | 0.55 | 0.1432 | predicted transporter |  |
| E100004130 | *b4242* | *mgtA* | 0.55 | 0.1734 | magnesium transporter | σ^70^ |
| E100002040 | *b2081* | *yegQ* | 0.55 | 0.1282 | predicted peptidase |  |
| E100001746 | *b1778* | *yeaA* | 0.55 | 0.1108 | methionine sulfoxide reductase B |  |
| E100000988 | *b1014* | *putA* | 0.54 | 0.1893 | fused DNA-binding transcriptional repressor | σ^70^ |
| E100002265 | *b2307* | *hisM* | 0.54 | 0.1861 | histidine/lysine/arginine/ornithine transporter subunit |  |
| E200001439 | *b1495* | *yddB* | 0.54 | 0.2146 | predicted porin protein |  |
| E100001136 | *b1163* | *ycgF* | 0.54 | 0.1811 | predicted FAD-binding phosphodiesterase |  |
| E100001353 | *b1382* | *ynbE* | 0.53 | 0.1641 | predicted lipoprotein |  |
| E200002802 | *b3727* | *pstC* | 0.53 | 0.1844 | phosphate transporter subunit |  |
| E100001305 | *b1334* | *fnr* | 0.53 | 0.0258 | DNA-binding transcriptional dual regulator, global regulator of anaerobic growth | σ^70^ |
| E200001137 | *b1055* | *yceA* | 0.53 | 0.1442 | hypothetical protein |  |
| E200001472 | *b1649* | *ydhM* | 0.52 | 0.2313 | predicted DNA-binding transcriptional regulator | σ^70^ |
| E100000704 | *b0721* | *sdhC* | 0.51 | 0.2073 | succinate dehydrogenase cytochrome b556 large membrane subunit | σ^70^ |
| E100002482 | *b2531* | *iscR* | 0.50 | 0.1589 | transcriptional dual regulator | σ^70^ |
| E200002834 | *b3820* | *yigI* | 0.50 | 0.3162 | hypothetical protein |  |
| E100001406 | *b1435* | *ydcP* | 0.50 | 0.2375 | predicted peptidase |  |
| E100000566 | *b0576* | *pheP* | 0.49 | 0.2122 | phenylalanine transporter | σ^70^ |
| E100001137 | *b1164* | *ycgZ* | 0.48 | 0.1164 | hypothetical protein |  |
| E100003525 | *b3603* | *lldP* | 0.48 | 0.2141 | L-lactate permease | σ^70^ |
| E100001386 | *b1415* | *aldA* | 0.48 | 0.2329 | aldehyde dehydrogenase A, NAD-linked | σ^70^ |
| E100003866 | *b3962* | *udhA* | 0.47 | 0.2392 | soluble pyridine nucleotide transhydrogenase | unknown |
| E100000705 | *b0722* | *sdhD* | 0.47 | 0.1783 | succinate dehydrogenase cytochrome b556 small membrane subunit | σ^70^ |
| E100000067 | *b0067* | *thiP* | 0.46 | 0.2603 | thiamin ABC transporter membrane protein |  |
| E100001773 | *b1805* | *fadD* | 0.46 | 0.2478 | acyl-CoA synthase | σ^70^ |
| E100001303 | *b1332* | *ynaJ* | 0.45 | 0.0100 | predicted inner membrane protein |  |
| E100003730 | *b3820* | *yigI* | 0.45 | 0.1769 | hypothetical protein |  |
| E100003001 | b3067 | rpoD | 0,45 | 0,2352 | RNA polymerase sigma factor | σ^24^,  σ^32^,  σ^70^ |
| E100000810 | *b0834* | *yliF* | 0.45 | 0.1933 | predicted diguanylate cyclase |  |
| E100003777 | *b3873* | *yihM* | 0.43 | 0.1640 | predicted sugar phosphate isomerase |  |
| E100003956 | *b4064* | *yjcD* | 0.43 | 0.2529 | predicted permease |  |
| E100001138 | *b1165* | *ymgA* | 0.42 | 0.1063 | hypothetical protein |  |
| E100001881 | *b1916* | *sdiA* | 0.40 | 0.2366 | DNA-binding transcriptional activator |  |
| E100000032 | *b0032* | *carA* | 0.39 | 0.1593 | carbamoyl-phosphate synthase small subunit | σ^70^ |
| E100000809 | *b0833* | *yliE* | 0.39 | 0.1651 | conserved inner membrane protein |  |
| E100001403 | *b1432* | *ydcM* | 0.38 | 0.2298 | predicted transposase |  |
| E200002658 | *b3603* | *lldP* | 0.35 | 0.1855 | L-lactate permease | σ^70^ |
| E100002874 | *b2939* | *yqgB* | 0.33 | 0.2029 | hypothetical protein |  |
| E100001028 | *b1055* | *yceA* | 0.31 | 0.1918 | hypothetical protein |  |
| E100001393 | *b1422* | *ydcI* | 0.31 | 0.2246 | predicted DNA-binding transcriptional regulator |  |
| E100002488 | *b2537* | *hcaR* | 0.29 | 0.2103 | DNA-binding transcriptional activator of 3-phenylpropionic acid catabolism | unknown |
| E100003025 | *b3092* | *uxaC* | 0.27 | 0.2405 | glucuronate isomerase | σ^70^ |
| E100003334 | *b3411* | *yhgA* | 0.25 | 0.2584 | predicted transposase |  |
| E100003576 | *b3654* | *yicE* | 0.23 | 0.2290 | predicted transporter |  |
