# Supplementary material for: Depletion of the non-coding regulatory 6S RNA in E. coli causes a surprising reduction in the expression of the translation machinery
Source: BMC Genomics. 2010 Mar 11;11:165. doi: 10.1186/1471-2164-11-165 (PMC2848244; doi:10.1186/1471-2164-11-165)
Supplement: Additional file 4 — Differentially expressed genes during stationary growth. The table lists all genes >1.5-fold differentially expressed in the DNA microarray analysis comparing the ssrS- strain MM139 with the wild type MC4100 during stationary growth. [file 1471-2164-11-165-S4.DOC]

**Table S3:** Genes >1.5-fold differentially expressed in the DNA microarray analysis comparing the transcriptome of the *ssrS*^-^ strain MM139 with the wild type MC4100 in early stationary phase. The relative mRNA values represent mean of 3 biological replicates including a color-swap (4 hybridisations).

| AROS™ V2.0 K12 DNA Oligo | b# | Gene | mRNA level *ssrS*^-^ / Wt | p-value | Annotation | Promotors according to Regulon DB [33] |
| --- | --- | --- | --- | --- | --- | --- |
| E100001655 | *b1687* | *ydiJ* | 4.53 | 0.1357 | predicted FAD-linked oxidoreductase |  |
| E100003812 | *b3908* | *sodA* | 4.09 | 0.1803 | superoxide dismutase, Mn | σ^70^ |
| E100001137 | *b1164* | *ycgZ* | 3.39 | 0.1937 | hypothetical protein |  |
| E100002567 | *b2620* | *smpB* | 3.13 | 0.1108 | SsrA-binding protein |  |
| E100003832 | *b3928* | *yiiU* | 2.95 | 0.0594 | hypothetical protein |  |
| E100003676 | *b3755* | *yieP* | 2.94 | 0.1981 | predicted transcriptional regulator |  |
| E100000893 | *b0918* | *kdsB* | 2.93 | 0.1647 | 3-deoxy-manno-octulosonate cytidylyltransferase |  |
| E100005367 | *b4522* | *yciX_1* | 2.83 | 0.1184 | hypothetical protein |  |
| E100002533 | *b2582* | *trxC* | 2.79 | 0.1508 | thioredoxin 2 | σ^70^ |
| E200002177 | *b2883* | *guaD* | 2.78 | 0.1243 | guanine deaminase |  |
| E100001224 | *b1253* | *yciA* | 2.77 | 0.1064 | predicted hydrolase |  |
| E100000031 | *b0031* | *dapB* | 2.73 | 0.1910 | dihydrodipicolinate reductase | σ^70^ |
| E200000053 | *b1111* | *ycfQ* | 2.70 | 0.1566 | predicted DNA-binding transcriptional regulator |  |
| E100000738 | *b0762* | *ybhT* | 2.60 | 0.0455 | hypothetical protein |  |
| E100002584 | *b2638* | *yfjU* | 2.60 | 0.2019 | CP4-57 prophage; conserved protein |  |
| E100000452 | *b0461* | *ybaJ* | 2.58 | 0.0052 | hypothetical protein | σ^70^ |
| E100003684 | *b3770* | *ilvE* | 2.51 | 0.1023 | branched-chain amino acid aminotransferase | unknown |
| E100002691 | *b2751* | *cysN* | 2.45 | 0.1420 | sulfate adenylyltransferase subunit 1 |  |
| E100003156 | *b3226* | *nanR* | 2.41 | 0.0515 | transcriptional regulator NanR |  |
| E100001419 | *b1448* | *yncA* | 2.40 | 0.0535 | predicted acyltransferase with acyl-CoA N-acyltransferase domain |  |
| E100004218 | *b4331* | *kptA* | 2.38 | 0.1296 | RNA 2'-phosphotransferase-like protein |  |
| E100002615 | *b2670* | *ygaW* | 2.35 | 0.0100 | predicted inner membrane protein |  |
| E100000451 | *b0460* | *hha* | 2.34 | 0.0156 | modulator of gene expression, with H-NS |  |
| E100003216 | *b3293* | *yhdN* | 2.25 | 0.1319 | hypothetical protein | σ^32^ |
| E100000227 | *b0234* | *yafP* | 2.15 | 0.2446 | predicted acyltransferase with acyl-CoA N-acyltransferase domain |  |
| E100003117 | *b3187* | *ispB* | 2.12 | 0.0852 | octaprenyl diphosphate synthase |  |
| E100004238 | *b4351* | *mrr* | 2.10 | 0.1270 | methylated adenine and cytosine restriction protein |  |
| E100003279 | *b3356* | *yhfA* | 2.10 | 0.0951 | hypothetical protein | σ^70^ |
| E200002699 | *b4294* | *insA-7* | 2.07 | 0.1447 | KpLE2 phage-like element; IS1 repressor protein InsA |  |
| E100001243 | *b1272* | *sohB* | 2.04 | 0.0054 | predicted inner membrane peptidase | σ^38^,  σ^70^ |
| E200000974 | *b0887* | *cydD* | 2.00 | 0.0630 | fused cysteine transporter subunits of ABC | unknown |
| E200002000 | *b2619* | *yfjG* | 2.00 | 0.0819 | hypothetical protein |  |
| E100002685 | *b2745* | *ygbO* | 1.97 | 0.1929 | tRNA pseudouridine synthase D |  |
| E100002394 | *b2443* | *yffL* | 1.96 | 0.0991 | CPZ-55 prophage; predicted protein |  |
| E100000657 | *b0674* | *asnB* | 1.94 | 0.0994 | asparagine synthetase B | unknown |
| E100001368 | *b1397* | *paaJ* | 1.91 | 0.0913 | acetyl-CoA acetyltransferase |  |
| E100000947 | *b0973* | *hyaB* | 1.91 | 0.1140 | hydrogenase 1, large subunit |  |
| E100000410 | *b0418* | *pgpA* | 1.89 | 0.0391 | phosphatidylglycerophosphatase A |  |
| E100002611 | *b2666* | *yqaE* | 1.87 | 0.1366 | predicted membrane protein |  |
| E200000222 | *b0418* | *pgpA* | 1.86 | 0.0563 | phosphatidylglycerophosphatase A |  |
| E100002607 | *b2662* | *gabT* | 1.84 | 0.1901 | 4-aminobutyrate aminotransferase |  |
| E100001595 | *b1625* | *ydgT* | 1.82 | 0.0427 | predicted regulator |  |
| E100002883 | *b2948* | *yqgE* | 1.82 | 0.1415 | hypothetical protein |  |
| E100000777 | *b0801* | *ybiC* | 1.82 | 0.0045 | predicted dehydrogenase |  |
| E100002635 | *b2690* | *yqaB* | 1.81 | 0.0981 | predicted hydrolase |  |
| E100003273 | *b3350* | *kefB* | 1.80 | 0.1029 | glutathione-regulated potassium-efflux system protein |  |
| E100002447 | *b2496* | *hda* | 1.79 | 0.2048 | DNA replication initiation factor | unknown |
| E100002300 | *b2342* | *yfcY* | 1.77 | 0.1547 | acetyl-CoA acetyltransferase | σ^70^ |
| E100003949 | *b4057* | *yjbR* | 1.77 | 0.0226 | hypothetical protein |  |
| E100002093 | *b2134* | *pbpG* | 1.77 | 0.1060 | D-alanyl-D-alanine endopeptidase |  |
| E100003895 | *b3999* | *yjaG* | 1.76 | 0.0807 | hypothetical protein |  |
| E100000759 | *b0783* | *moaC* | 1.76 | 0.1678 | molybdenum cofactor biosynthesis protein C |  |
| E200001999 | *b2617* | *smpA* | 1.75 | 0.0795 | small membrane lipoprotein | σ^24^ |
| E100000001 | *b0001* | *thrL* | 1.73 | 0.0497 | thr operon leader peptide | σ^70^ |
| E200000852 | *b0710* | *ybgI* | 1.73 | 0.0481 | conserved metal-binding protein | unknown |
| E100002801 | *b2865* | *ygeR* | 1.72 | 0.0849 | Tetratricopeptide repeat transcriptional regulator |  |
| E100003364 | *b3441* | *yhhY* | 1.71 | 0.0435 | predicted acetyltransferase | σ^70^ |
| E100003388 | *b3465* | *yhhF* | 1.71 | 0.0687 | predicted methyltransferase |  |
| E100003770 | *b3866* | *yihI* | 1.70 | 0.0508 | hypothetical protein |  |
| E100000406 | *b0414* | *ribD* | 1.70 | 0.0619 | fused diaminohydroxyphosphoribosylaminopyrimidine deaminase and 5-amino-6-(5-phosphoribosylamino) uracil reductase |  |
| E100000407 | *b0415* | *ribH* | 1.69 | 0.2082 | riboflavin synthase subunit beta | σ^32^ |
| E100001407 | *b1436* | *yncJ* | 1.69 | 0.0713 | hypothetical protein |  |
| E100004003 | *b4111* | *proP* | 1.69 | 0.0997 | proline/glycine betaine transporter | σ^38^,  σ^70^ |
| E100000119 | *b0119* | *yacL* | 1.68 | 0.0023 | hypothetical protein | unknown |
| E200000352 | *b2023* | *hisH* | 1.68 | 0.0402 | imidazole glycerol phosphate synthase subunit HisH |  |
| E100000405 | *b0413* | *ybaD* | 1.68 | 0.0761 | hypothetical protein | σ^70^ |
| E100002606 | *b2661* | *gabD* | 1.68 | 0.0513 | succinate-semialdehyde dehydrogenase I, NADP-dependent | σ^38^,  σ^70^ |
| E100002301 | *b2343* | *yfcZ* | 1.67 | 0.0802 | hypothetical protein |  |
| E100000520 | *b0529* | *folD* | 1.67 | 0.1306 | bifunctional 5,10-methylene-tetrahydrofolate dehydrogenase/ 5,10-methylene-tetrahydrofolate cyclohydrolase | unknown |
| E100003947 | *b4055* | *aphA* | 1.67 | 0.0763 | acid phosphatase/phosphotransferase, class B, non-specific |  |
| E100000080 | *b0080* | *fruR* | 1.67 | 0.0175 | DNA-binding transcriptional dual regulator |  |
| E100002629 | *b2684* | *mprA* | 1.67 | 0.1590 | DNA-binding transcriptional repressor of microcin B17 synthesis and multidrug efflux | σ^70^ |
| E100003093 | *b3161* | *mtr* | 1.66 | 0.0453 | tryptophan transporter of high affinity | σ^70^, unknown |
| E100003831 | *b3927* | *glpF* | 1.66 | 0.1510 | glycerol facilitator | σ^70^ |
| E200000073 | *b1477* | *yddM* | 1.66 | 0.0972 | predicted DNA-binding transcriptional regulator |  |
| E100000894 | *b0919* | *ycbJ* | 1.66 | 0.0185 | hypothetical protein |  |
| E100002767 | *b2830* | *ygdP* | 1.66 | 0.0701 | dinucleoside polyphosphate hydrolase |  |
| E100000931 | *b0956* | *ycbG* | 1.65 | 0.0500 | hypothetical protein | unknown |
| E100002101 | *b2142* | *yohK* | 1.65 | 0.1821 | predicted inner membrane protein |  |
| E200003028 | *b4124* | *dcuR* | 1.65 | 0.2183 | DNA-binding response regulator in two-component regulatory system with DcuS | σ^70^ |
| E200001357 | *b1278* | *pgpB* | 1.65 | 0.0396 | phosphatidylglycerophosphatase B |  |
| E200000246 | *b4284 b0256 b1404 b4492* | *insI-3, insI-1, insI-2, ydbA* | 1.65 | 0.0527 | KpLE2 phage-like element; IS30 transposase | CP4-6 prophage; IS30 transposase | IS30 transposase | - |  |
| E200002992 | *b4057* | *yjbR* | 1.65 | 0.0519 | hypothetical protein |  |
| E100003266 | *b3343* | *yheL* | 1.64 | 0.0695 | predicted intracellular sulfur oxidation protein |  |
| E100004100 | *b4212* | *ytfH* | 1.64 | 0.2468 | predicted transcriptional regulator |  |
| E100000803 | *b0827* | *moeA* | 1.64 | 0.1411 | molybdopterin biosynthesis protein | σ^70^ |
| E100003768 | *b3863* | *polA* | 1.63 | 0.0441 | DNA polymerase I | unknown |
| E100003948 | *b4056* | *yjbQ* | 1.63 | 0.0357 | hypothetical protein |  |
| E100002289 | *b2331* | *yfcN* | 1.63 | 0.0536 | hypothetical protein |  |
| E100004277 | *b4393* | *trpR* | 1.63 | 0.0297 | Trp operon repressor | σ^70^ |
| E100004443 | *b4409* | *blr* | 1.62 | 0.2176 | beta-lactam resistance membrane protein |  |
| E100003390 | *b3467* | *yhhM* | 1.62 | 0.0929 | hypothetical protein |  |
| E200000219 | *b0384* | *psiF* | 1.62 | 0.1639 | hypothetical protein |  |
| E200000161 | *b4252* | *yjgK* | 1.62 | 0.1898 | hypothetical protein |  |
| E100004094 | *b4206* | *ytfB* | 1.62 | 0.0514 | predicted cell envelope opacity-associated protein |  |
| E200001361 | *b1285* | *gmr* | 1.62 | 0.1028 | modulator of Rnase II stability | σ^70^ |
| E100001199 | *b1226* | *narJ* | 1.61 | 0.1170 | molybdenum-cofactor-assembly chaperone subunit delta subunit) of nitrate reductase 1 |  |
| E100003022 | *b3089* | *sstT* | 1.61 | 0.0317 | sodium:serine/threonine symporter | unknown |
| E100000402 | *b0410* | *yajD* | 1.61 | 0.0111 | hypothetical protein |  |
| E200001328 | *b1214* | *ychA* | 1.61 | 0.1921 | predicted transcriptional regulator |  |
| E100004120 | *b4232* | *fbp* | 1.60 | 0.0963 | fructose-1,6-bisphosphatase |  |
| E100000933 | *b0958* | *sulA* | 1.60 | 0.0165 | SOS cell division inhibitor | σ^70^ |
| E100002477 | *b2526* | *hscA* | 1.59 | 0.2708 | chaperone protein HscA | unknown |
| E100000843 | *b0867* | *ybjR* | 1.59 | 0.1508 | predicted amidase and lipoprotein |  |
| E100000857 | *b0881* | *clpS* | 1.59 | 0.0111 | ATP-dependent Clp protease adaptor protein ClpS |  |
| E100003331 | *b3408* | *feoA* | 1.59 | 0.2142 | ferrous iron transport protein A | σ^70^ |
| E100000682 | *b0699* | *ybfA* | 1.58 | 0.0376 | hypothetical protein |  |
| E200002473 | *b3279* | *yrdA* | 1.57 | 0.0475 | hypothetical protein | σ^32^ |
| E100002565 | *b2618* | *yfjF* | 1.57 | 0.1271 | hypothetical protein |  |
| E200000414 | *b3467* | *yhhM* | 1.57 | 0.1945 | hypothetical protein |  |
| E100000353 | *b2860, b1996, b1402, b4273, b0361, b4579, b3045,| b1578* | *insD-4, insD-3, insD-2, insD-6, insD-1, yaiX, insD-5, insD-7* | 1.57 | 0.2030 | IS2 insertion element transposase InsAB' | CP4-44 prophage; IS2 insertion element transposase InsAB' | IS2 insertion element transposase InsAB' | KpLE2 phage-like element; IS2 insertion element transposase InsAB' | IS2 insertion element transposase In |  |
| E100002637 | *b2697* | *alaS* | 1.56 | 0.2096 | alanyl-tRNA synthetase | unknown |
| E100003860 | *b3956* | *ppc* | 1.56 | 0.0189 | phosphoenolpyruvate carboxylase | unknown |
| E100002287 | *b2329* | *aroC* | 1.56 | 0.1090 | chorismate synthase |  |
| E100002167 | *b2209* | *eco* | 1.56 | 0.1620 | ecotin precursor |  |
| E100003459 | *b3536* | *bcsE* | 1.56 | 0.0340 | hypothetical protein |  |
| E100000806 | *b0830* | *yliB* | 1.56 | 0.2985 | predicted peptide transporter subunit: periplasmic-binding component of ABC superfamily |  |
| E100000758 | *b0782* | *moaB* | 1.56 | 0.1113 | molybdopterin biosynthesis protein B | unknown |
| E100003691 | *b3777* | *yifN* | 1.56 | 0.1411 | conserved protein (pseudogene) |  |
| E100003185 | *b3255* | *accB* | 1.55 | 0.0391 | acetyl-CoA carboxylase | σ^70^ |
| E100001809 | *b1841* | *yobA* | 1.55 | 0.0707 | hypothetical protein |  |
| E100004056 | *b4168* | *yjeE* | 1.55 | 0.0948 | ATPase with strong ADP affinity |  |
| E100000203 | *b0209* | *yafD* | 1.55 | 0.0622 | hypothetical protein | σ^32^ |
| E100000882 | *b0907* | *serC* | 1.55 | 0.1291 | phosphoserine aminotransferase | σ^70^ |
| E200002549 | *b3438* | *gntR* | 1.55 | 0.1950 | DNA-binding transcriptional repressor | σ^70^, unknown |
| E100000696 | *b0713* | *ybgL* | 1.55 | 0.0403 | hypothetical protein |  |
| E100000671 | *b0688* | *pgm* | 1.55 | 0.1135 | phosphoglucomutase |  |
| E100000134 | *b0134* | *panB* | 1.55 | 0.0459 | 3-methyl-2-oxobutanoate hydroxymethyltransferase | σ^70^ |
| E100000221 | *b0228* | *yafM* | 1.54 | 0.0250 | hypothetical protein |  |
| E100005853 | *b4558* | *yifL* | 1.54 | 0.0043 | predicted lipoprotein | σ^70^ |
| E100000497 | *b0506* | *allR* | 1.54 | 0.0725 | DNA-binding transcriptional repressor | unknown |
| E100004067 | *b4179* | *rnr* | 1.54 | 0.0001 | exoribonuclease R, RNase R |  |
| E100003754 | *b3844* | *fre* | 1.53 | 0.1005 | NAD(P)H-flavin reductase | unknown |
| E100003260 | *b3337* | *bfd* | 1.52 | 0.1524 | bacterioferritin-associated ferredoxin |  |
| E200000827 | *b0674* | *asnB* | 1.52 | 0.0602 | asparagine synthetase B | unknown |
| E100001612 | *b1642* | *slyA* | 1.52 | 0.0388 | transcriptional regulator SlyA |  |
| E100002001 | *b2042* | *galF* | 1.52 | 0.1871 | predicted subunit with GalU | unknown |
| E100002347 | *b2390* | *ypeC* | 1.52 | 0.1287 | hypothetical protein |  |
| E100003018 | *b3085* | *ygjP* | 1.52 | 0.0576 | predicted metal dependent hydrolase |  |
| E200000119 | *b2431* | *yfeX* | 1.52 | 0.1942 | hypothetical protein |  |
| E100003577 | *b3655* | *yicH* | 1.52 | 0.0445 | hypothetical protein |  |
| E100000695 | *b0712* | *ybgK* | 1.52 | 0.0019 | predicted enzyme subunit |  |
| E100002692 | *b2752* | *cysD* | 1.51 | 0.1881 | sulfate adenylyltransferase subunit 2 | σ^70^ |
| E200000847 | *b0695* | *kdpD* | 1.51 | 0.0365 | fused sensory histidine kinase in two-component regulatory system with KdpE: signal sensing protein |  |
| E100003430 | *b3507* | *yhiF* | 1.51 | 0.0042 | predicted DNA-binding ranscriptional regulator |  |
| E100001160 | *b1187* | *fadR* | 1.51 | 0.0174 | fatty acid metabolism regulator | unknown |
| E100001993 | *b2034* | *wbbI* | 1.51 | 0.1884 | hypothetical protein |  |
| E100003105 | *b3175* | *secG* | 1.51 | 0.0404 | protein-export membrane protein | σ^70^, |
| E100002363 | *b2412* | *zipA* | 1.51 | 0.0054 | cell division protein ZipA |  |
| E100002530 | *b2579* | *yfiD* | 1.50 | 0.0781 | pyruvate formate lyase subunit |  |
| E100001541 | *b15*^70^ | *dicA* | 1.50 | 0.0839 | Qin prophage; predicted regulator for DicB |  |
| E100000027 | *b0027* | *lspA* | 1.50 | 0.1199 | signal peptidase II | σ^70^ |
| E100003232 | *b3309* | *rplX* | 0.66 | 0.0159 | 50S ribosomal protein L24 |  |
| E100003264 | *b3341* | *rpsG* | 0.66 | 0.0075 | 30S ribosomal protein S7 |  |
| E1000011^70^ | *b1197* | *treA* | 0.66 | 0.0249 | periplasmic trehalase | σ^38^ |
| E100002885 | *b2950* | *yggR* | 0.66 | 0.1952 | predicted transporter |  |
| E100003840 | *b3936* | *rpmE* | 0.66 | 0.1179 | 50S ribosomal subunit protein L31 | σ^32^ |
| E100000572 | *b0582 b2394 b0016* | *insL-2 insL-| insL-1* | 0.66 | 0.0085 | IS186/IS421 transposase | IS186/IS421 transposase | IS186/IS421 transposase |  |
| E100004115 | *b4227* | *ytfQ* | 0.66 | 0.0051 | predicted sugar transporter subunit: periplasmic-binding component of ABC superfamily |  |
| E100002556 | *b2609* | *rpsP* | 0.66 | 0.0482 | 30S ribosomal protein S16 | σ^70^ |
| E100003437 | *b3514* | *mdtF* | 0.66 | 0.0132 | multidrug transporter, RpoS-dependent |  |
| E100001860 | *b1892* | *flhD* | 0.66 | 0.1067 | transcriptional activator FlhD | σ^70^ |
| E100000605 | *b0615* | *citF* | 0.65 | 0.0245 | citrate lyase, citrate-ACP transferase (alpha) subunit |  |
| E100001878 | *b1913* | *uvrC* | 0.65 | 0.2734 | excinuclease ABC subunit C | σ^70^ |
| E100004090 | *b4202* | *rpsR* | 0.65 | 0.0004 | 30S ribosomal protein S18 |  |
| E100000473 | *b0482* | *ybaP* | 0.65 | 0.0977 | hypothetical protein |  |
| E100003233 | *b3310* | *rplN* | 0.65 | 0.0056 | 50S ribosomal protein L14 | unknown |
| E100003528 | *b3606* | *yibK* | 0.65 | 0.0505 | predicted rRNA methylase |  |
| E100000512 | *b0521* | *ybcF* | 0.65 | 0.1132 | predicted carbamate kinase |  |
| E100001798 | *b1830* | *prc* | 0.65 | 0.1785 | carboxy-terminal protease for penicillin-binding protein 3 |  |
| E100003219 | *b3296* | *rpsD* | 0.65 | 0.0694 | 30S ribosomal protein S4 |  |
| E100002144 | *b2185* | *rplY* | 0.65 | 0.0434 | 50S ribosomal protein L25 |  |
| E100004088 | *b4200* | *rpsF* | 0.64 | 0.0395 | 30S ribosomal protein S6 | unknown |
| E100001098 | *b1125* | *potB* | 0.64 | 0.0988 | spermidine/putrescine ABC transporter membrane protein |  |
| E200002605 | *b3513* | *mdtE* | 0.64 | 0.1250 | multidrug resistance efflux transporter | unknown |
| E100000613 | *b0623* | *cspE* | 0.64 | 0.0220 | cold shock protein E | σ^70^ |
| E100002929 | *b2995* | *hybB* | 0.64 | 0.0294 | predicted hydrogenase 2 cytochrome b type component |  |
| E100002208 | *b2250* | *yfaZ* | 0.64 | 0.0189 | predicted outer membrane porin protein |  |
| E100000831 | *b0855* | *potG* | 0.64 | 0.2073 | putrescine transporter subunit: ATP-binding component of ABC superfamily |  |
| E100003962 | *b40*^70^ | *nrfA* | 0.64 | 0.3116 | nitrite reductase, formate-dependent, cytochrome | σ^70^ |
| E100000942 | *b0967* | *yccW* | 0.64 | 0.1060 | predicted methyltransferase |  |
| E100001999 | *b2040* | *rfbD* | 0.64 | 0.0955 | dTDP-4-dehydrorhamnose reductase subunit, NAD(P)-binding, of dTDP-L-rhamnose synthase |  |
| E100003673 | *b3752* | *rbsK* | 0.64 | 0.0016 | ribokinase |  |
| E100003265 | *b3342* | *rpsL* | 0.64 | 0.0339 | 30S ribosomal protein S12 | unknown |
| E100001488 | *b1517* | *yneB* | 0.64 | 0.2181 | hypothetical protein |  |
| E100002882 | *b2947* | *gshB* | 0.63 | 0.2639 | glutathione synthetase |  |
| E100001305 | *b1334* | *fnr* | 0.63 | 0.0096 | DNA-binding transcriptional dual regulator, global regulator of anaerobic growth | σ^70^ |
| E100003697 | *b3783* | *rho* | 0.63 | 0.0647 | transcription termination factor Rho |  |
| E100003296 | *b4474* | *frlC* | 0.63 | 0.1545 | predicted isomerase |  |
| E200001989 | *b2587* | *kgtP* | 0.63 | 0.1416 | alpha-ketoglutarate transporter |  |
| E100000904 | *b0929* | *ompF* | 0.63 | 0.0475 | outer membrane porin 1a (Ia;b;F) | σ^70^ |
| E100002520 | *b2569* | *lepA* | 0.62 | 0.0522 | GTP-binding protein LepA | unknown |
| E200000046 | *b1562* | *hokD* | 0.62 | 0.0537 | Qin prophage; small toxic polypeptide |  |
| E100004222 | *b4335* | *yjiM* | 0.62 | 0.0739 | predicted 2-hydroxyglutaryl-CoA dehydratase |  |
| E100003161 | *b3231* | *rplM* | 0.62 | 0.0428 | 50S ribosomal protein L13 | σ^70^ |
| E100002989 | *b3055* | *htrG* | 0.62 | 0.0704 | predicted signal transduction protein (SH3 domain) | σ^24^ |
| E100001678 | *b1710* | *btuE* | 0.62 | 0.1035 | predicted glutathione peroxidase |  |
| E100000622 | *b0632* | *dacA* | 0.62 | 0.1825 | D-alanyl-D-alanine carboxypeptidase penicillin-binding protein 5) |  |
| E100002682 | *b2742* | *nlpD* | 0.62 | 0.1055 | predicted outer membrane lipoprotein | unknown |
| E100003263 | *b3340* | *fusA* | 0.62 | 0.0120 | elongation factor EF-2 | σ^24^ |
| E100000398 | *b0406* | *tgt* | 0.62 | 0.0486 | queuine tRNA-ribosyltransferase | unknown |
| E200001982 | *b2568* | *lepB* | 0.61 | 0.0195 | leader peptidase (signal peptidase I) |  |
| E100002737 | *b2797* | *sdaB* | 0.61 | 0.0472 | L-serine deaminase II |  |
| E100003764 | *b3859* | *yihE* | 0.61 | 0.0267 | predicted kinase | σ^70^ |
| E100003765 | *b3860* | *dsbA* | 0.61 | 0.0180 | periplasmic protein disulfide isomerase I | σ^70^ |
| E100003261 | *b3338* | *chiA* | 0.61 | 0.2503 | periplasmic endochitinase | σ^70^ |
| E100002813 | *b2877* | *ygfJ* | 0.60 | 0.0172 | hypothetical protein |  |
| E100002108 | *b2149* | *mglA* | 0.60 | 0.0263 | fused methyl-galactoside transporter subunits of ABC superfamily: ATP-binding components | unknown |
| E100000978 | *b1004* | *wrbA* | 0.60 | 0.0777 | TrpR binding protein WrbA | unknown |
| E100002852 | *b2917* | *sbm* | 0.60 | 0.0716 | methylmalonyl-CoA mutase | unknown |
| E100001891 | *b1926* | *fliT* | 0.60 | 0.1452 | predicted chaperone |  |
| E100001304 | *b1333* | *uspE* | 0.59 | 0.0409 | stress-induced protein | unknown |
| E100000642 | *b0652* | *gltL* | 0.59 | 0.1803 | glutamate and aspartate transporter subunit |  |
| E100001097 | *b1124* | *potC* | 0.59 | 0.1196 | spermidine/putrescine ABC transporter membrane protein |  |
| E100002323 | *b2366* | *dsdA* | 0.59 | 0.0038 | D-serine dehydratase | σ^70^ |
| E100002816 | *b2880* | *ygfM* | 0.58 | 0.0565 | predicted oxidoreductase |  |
| E100001453 | *b1482* | *osmC* | 0.58 | 0.0356 | osmotically inducible, stress-inducible membrane protein | σ^38^,  σ^70^ |
| E100001457 | *b1486* | *ddpB* | 0.58 | 0.0760 | D-ala-D-ala transporter subunit |  |
| E100003879 | *b3983* | *rplK* | 0.58 | 0.0070 | 50S ribosomal protein L11 | unknown |
| E100002812 | *b2876* | *yqeC* | 0.58 | 0.0228 | hypothetical protein |  |
| E100002874 | *b2939* | *yqgB* | 0.58 | 0.1491 | hypothetical protein |  |
| E100001895 | *b1930* | *yedF* | 0.58 | 0.0794 | hypothetical protein |  |
| E100001558 | *b1588* | *ynfF* | 0.58 | 0.0886 | oxidoreductase subunit |  |
| E100001107 | *b1134* | *ymfB* | 0.57 | 0.1006 | bifunctional thiamin pyrimidine pyrophosphate hydrolase/ thiamin pyrophosphate hydrolase |  |
| E100003880 | *b3984* | *rplA* | 0.57 | 0.0384 | 50S ribosomal protein L1 |  |
| E100003234 | *b3311* | *rpsQ* | 0.57 | 0.3264 | 30S ribosomal protein S17 |  |
| E100002553 | *b2606* | *rplS* | 0.57 | 0.0231 | 50S ribosomal protein L19 |  |
| E100001252 | *b1281* | *pyrF* | 0.57 | 0.0689 | orotidine 5'-phosphate decarboxylase | σ^70^ |
| E100003838 | *b3934* | *cytR* | 0.56 | 0.0953 | DNA-binding transcriptional dual regulator | σ^70^ |
| E100002555 | *b2608* | *rimM* | 0.56 | 0.0159 | 16S rRNA-processing protein |  |
| E100002736 | *b2796* | *sdaC* | 0.56 | 0.0717 | predicted serine transporter | σ^70^ |
| E100002073 | *b2114* | *metG* | 0.56 | 0.0309 | methionyl-tRNA synthetase | unknown |
| E100001303 | *b1332* | *ynaJ* | 0.55 | 0.0074 | predicted inner membrane protein |  |
| E100003558 | *b3636* | *rpmG* | 0.55 | 0.0327 | 50S ribosomal protein L33 |  |
| E100000690 | *b0*^70^*7* | *ybgA* | 0.55 | 0.1859 | hypothetical protein | σ^38^ |
| E100000629 | *b0639* | *nadD* | 0.55 | 0.0578 | nicotinic acid mononucleotide adenyltransferase |  |
| E100000162 | *b0162* | *cdaR* | 0.54 | 0.1150 | DNA-binding transcriptional activator | unknown |
| E100004089 | *b4201* | *priB* | 0.54 | 0.0596 | primosomal replication protein N |  |
| E100000872 | *b0897* | *ycaC* | 0.54 | 0.0298 | predicted hydrolase |  |
| E100003884 | *b3988* | *rpoC* | 0.54 | 0.0160 | DNA-directed RNA polymerase subunit beta' |  |
| E100001991 | *b2032* | *wbbK* | 0.54 | 0.0917 | lipopolysaccharide biosynthesis protein |  |
| E200000012 | *b4423 | b4419 | b4421* | *ldrC ldrA ldrB* | 0.53 | 0.1442 | toxic polypeptide, small | "toxic polypeptide, small " | "toxic polypeptide, small " |  |
| E100003416 | *b3493* | *pitA* | 0.53 | 0.1045 | phosphate transporter, low-affinity | unknown |
| E100001278 | *b1307* | *pspD* | 0.53 | 0.2021 | peripheral inner membrane phage-shock protein |  |
| E100002657 | *b2717* | *hycI* | 0.53 | 0.2342 | protease involved in processing C-terminal end of HycE |  |
| E100003883 | *b3987* | *rpoB* | 0.53 | 0.0303 | DNA-directed RNA polymerase subunit beta | unknown |
| E100003441 | *b3518* | *yhjA* | 0.52 | 0.2340 | predicted cytochrome C peroxidase | σ^70^ |
| E100003477 | *b3555* | *yiaG* | 0.52 | 0.0072 | predicted transcriptional regulator | σ^38^ |
| E100004246 | *b4359* | *mdoB* | 0.51 | 0.1287 | phosphoglycerol transferase I |  |
| E200001810 | *b2148* | *mglC* | 0.51 | 0.0471 | beta-methylgalactoside transporter inner membrane component |  |
| E100001292 | *b1321* | *ycjX* | 0.51 | 0.2627 | conserved protein with nucleoside triphosphate hydrolase domain | σ^32^ |
| E100002806 | *b28*^70^ | *ygeW* | 0.51 | 0.0612 | hypothetical protein |  |
| E100002190 | *b2232* | *ubiG* | 0.50 | 0.1932 | 3-demethylubiquinone-9 3-methyltransferase | σ^70^ |
| E100003646 | *b3725* | *pstB* | 0.50 | 0.2180 | phosphate transporter subunit | σ^70^ |
| E100000867 | *b0892* | *ycaJ* | 0.50 | 0.1711 | recombination protein |  |
| E200000773 | *b0474* | *adk* | 0.49 | 0.0734 | adenylate kinase | unknown |
| E100002859 | *b2924* | *mscS* | 0.49 | 0.0013 | mechanosensitive channel | unknown |
| E100000555 | *b0565* | *ompT* | 0.48 | 0.0464 | DLP12 prophage; outer membrane protease VII outer membrane protein | unknown |
| E100002826 | *b2890* | *lysS* | 0.48 | 0.0117 | lysine tRNA synthetase, constitutive |  |
| E200002993 | *b4058* | *uvrA* | 0.48 | 0.2006 | excinuclease ABC subunit A | σ^70^ |
| E100000015 | *b0015* | *dnaJ* | 0.47 | 0.1056 | chaperone Hsp40, co-chaperone with DnaK |  |
| E100000844 | *b0868* | *ybjS* | 0.46 | 0.1648 | predicted NAD(P)H-binding oxidoreductase with NAD(P)-binding Rossmann-fold domain |  |
| E100003960 | *b4068* | *yjcH* | 0.46 | 0.1311 | conserved inner membrane protein involved in acetate transport |  |
| E100003624 | *b3*^70^*3* | *rpmH* | 0.45 | 0.1461 | 50S ribosomal protein L34 | unknown |
| E100001512 | *b1541* | *ydfZ* | 0.44 | 0.0103 | hypothetical protein |  |
| E100002817 | *b2881* | *xdhD* | 0.44 | 0.1839 | fused predicted xanthine/hypoxanthine oxidase: |  |
| E100000461 | *b04*^70^ | *dnaX* | 0.44 | 0.1981 | DNA polymerase III subunits gamma and tau | unknown |
| E100001^70^0 | *b1732* | *katE* | 0.42 | 0.0298 | hydroperoxidase HPII(III) (catalase) | σ^38^ |
| E200001811 | *b2149* | *mglA* | 0.42 | 0.0208 | fused methyl-galactoside transporter subunits of ABC superfamily: ATP-binding components | unknown |
| E100004101 | *b4213* | *cpdB* | 0.41 | 0.1711 | bifunctional 2',3'-cyclic nucleotide | σ^70^ |
| E200000873 | *b0772* | *ybhC* | 0.40 | 0.1434 | predicted pectinesterase | unknown |
| E100001725 | *b1757* | *ynjE* | 0.40 | 0.1132 | predicted thiosulfate sulfur transferase |  |
| E100000644 | *b0654* | *gltJ* | 0.37 | 0.2194 | glutamate and aspartate transporter subunit |  |
| E100003020 | *b3087* | *ygjR* | 0.37 | 0.2219 | predicted NAD(P)-binding dehydrogenase |  |
| E100002814 | *b2878* | *ygfK* | 0.35 | 0.0210 | predicted oxidoreductase, Fe-S subunit |  |
| E100001229 | *b1258* | *yciF* | 0.35 | 0.0306 | hypothetical protein |  |
| E100000926 | *b0951* | *pqiB* | 0.23 | 0.1713 | paraquat-inducible protein B |  |
| E100001206 | *b1235* | *rssB* | 0.11 | 0.2043 | response regulator of RpoS | σ^38^ |

Supplementary Table 3 had been prepared without applying the signal/noise filter in order to get a largely unbiased set of all genes, including those with very weak expression.
