# Supplementary material for: Depletion of the non-coding regulatory 6S RNA in E. coli causes a surprising reduction in the expression of the translation machinery
Source: BMC Genomics. 2010 Mar 11;11:165. doi: 10.1186/1471-2164-11-165 (PMC2848244; doi:10.1186/1471-2164-11-165)
Supplement: Additional file 5 — Comparison of selected miroarray data with qRT-PCR analysis. The table lists a comparison of DNA-microarray data and qRT-PCR analysis for selected genes in early stationary phase. [file 1471-2164-11-165-S5.DOC]

**Additional file 5**

**Table S4:** qRT-PCR analysis of two independent biological replicates I and II corroborate DNA-microarray data of *osmY*, *rpoD* and *bolA* when comparing the RNA levels of *E. coli* WT and 6S RNA deficient Mutant MM139 in early stationary phase. *rrsA* served as a control.

|  |  | | C_T_ ^a^ : | | | | Average Ratio *__^-^* _/WT_ | |
| --- | --- | --- | --- | --- | --- | --- | --- | --- |
|  |  | |  |  |  |  |  |  |
| gene |  | | WT_stat_ I | **^−^_stat_ I | WT_stat_ II | **^−^_stat_ II | qRT-PCR ^b^ | DNA-Microarrays |
|  |  | |  |  |  |  |  |  |
| *osmY* | | 15.99 ± 0.1 | | 15.81 ± 0.03 | 16.91 ± 0.12 | 16.48 ± 0.16 | 1.23 ± 0.1 | 1.13 ± 0.4 |
| *rpoD* |  | | 19.06 ± 0.12 | 19.17 ± 0.06 | 19.60 ± 0.14 | 19.98 ± 0.2 | 0.85 ± 0.08 | 0.85 ± 0.2 |
| *bolA* |  | | 18.24 ± 0.06 | 17.59 ± 0.02 | 18.61 ± 0.03 | 18.58 ± 0.1 | 1.28 ± 0.26 | 1.03 ± 0.03 |
| *rrsA* |  | | 3.24 ± 0.6 | 3.43 ± 0.04 | 3.87 ± 0.14 | 3.46 ± 0.25 | 1.1 ± 0.22 | not present |
|  |  | |  |  |  |  |  |  |

^a)^ Measured in duplicate for each gene. C_T_ is defined as the cycle at which fluorescence is determined to be statistically significantly above background and is inversely proportional to the log of the initial copy number. This value was calculated by the instrument software.

^b)^ Gene expression ratios were calculated based on C_T_ values using REST 2005 software
